# Emerging therapeutic targets in non-infectious uveitis and scleritis

**DOI:** 10.3389/fmed.2026.1846374

**Published:** 2026-05-15

**Authors:** Usanee Tungsattayathitthan, Debra A. Goldstein, Timothy M. Janetos

**Affiliations:** 1 Department of Ophthalmology, Feinberg School of Medicine, Northwestern University, Chicago, IL, United States; 2 Department of Ophthalmology, Faculty of Medicine Siriraj Hospital, Mahidol University, Bangkok, Thailand

**Keywords:** biologics, scleritis, therapeutics, treatment, uveitis

## Abstract

Non-infectious uveitis and scleritis are significant causes of visual impairment, and currently available targeted and effective therapeutic options remain limited. Multiple immunologic pathways are currently under investigation and targeted therapeutics have the potential to change the treatment landscape and improve visual outcomes. This review synthesizes potential upcoming local and systemic treatments for non-infectious uveitis and scleritis with a focus on therapeutics with human data, noting those with the most promising evidence, and areas of gaps in research.

## 1 Introduction

Uveitis comprises a heterogeneous set of intraocular inflammatory diseases with variable clinical manifestations and risk of visual impairment. Given the potential for irreversible vision loss, timely and effective therapeutic intervention is essential. The principal therapeutic objective in non-infectious uveitis (NIU) and non-infectious scleritis (NIS) management is to achieve rapid and sustained inflammatory control, prevent recurrence and structural complications, preserve visual function, and minimize treatment-related adverse effects.

The therapeutic landscape of ocular inflammatory disease has evolved with the introduction of biologic therapies, reflecting a shift from non-specific anti-inflammatory treatment toward selective immune pathway modulation. Tumor necrosis factor (TNF)-α inhibitors, including infliximab, adalimumab, and golimumab, represent the most extensively studied and commonly utilized biologic class to date in NIU and NIS. Among these, adalimumab is currently the only biologic approved by both the U.S. Food and Drug Administration (FDA) and the European Medicines Agency for the treatment of non-infectious intermediate, posterior, and panuveitis, with robust evidence supporting its efficacy in reducing relapse rates and preserving visual acuity (VA) (1). In contrast, no biologic agents are currently approved for NIS. However, anti-TNF-α agents, especially infliximab and adalimumab, have shown favorable efficacy in refractory scleritis (2, 3). In recent years, anti-TNF-α therapy has been introduced earlier in the therapeutic course and is increasingly considered as first-line therapy in selected clinical settings, supported by clinical trial data demonstrating more rapid corticosteroid-sparing effects and higher rates of steroid discontinuation compared with conventional immunosuppressive therapy (4).

Despite these advances, biologic agents do not uniformly achieve durable remission in real-world practice. Even with anti-TNF-α therapy, inflammatory complications such as macular edema may still occur (5). Furthermore, monoclonal antibody (mAb)-based biologics require parenteral administration, exhibit limited tissue penetration, and may lose efficacy over time due to immunogenicity. Additionally, given the marked heterogeneity of NIU and NIS subtypes, treatment responses vary considerably, and relapse remains common. These challenges underscore the need for more selective, mechanism-based therapeutic strategies targeting specific immune pathways involved in ocular inflammation. A growing number of emerging therapeutic agents, including novel biologics, small-molecule inhibitors, and targeted immune modulators, have been developed and evaluated in clinical studies. This review aims to synthesize current evidence on emerging therapeutic targets in NIU and NIS, with a particular focus on agents investigated in human clinical trials.

### 1.1 Method of literature search

A comprehensive literature search was conducted to identify clinical studies evaluating emerging therapeutic targets in NIU and NIS. Searches were performed in PubMed and included articles published between January 2004 and 31 March 2026. Search terms were applied using Medical Subject Headings (MeSH) and free-text keywords.

Disease-related search terms included “Uveitis” (MeSH), uveitis, “Scleritis” (MeSH), scleritis, and ocular inflammation. These terms were combined with therapy-specific keywords representing both individual drug names and mechanism-based descriptors. Cytokine-targeted therapies were identified using terms related to interleukin (IL) blockade, including IL-1 inhibitors (anakinra, canakinumab, gevokizumab), IL-6 inhibitors (tocilizumab, sarilumab, vamikibart), IL-17 inhibitors (secukinumab, ixekizumab, guselkumab), and IL-12/23 inhibition (ustekinumab). Intracellular signaling and small-molecule pathways were captured using terms related to Janus kinase (JAK) inhibition (tofacitinib, baricitinib, upadacitinib, filgotinib, brepocitinib), dihydroorotate dehydrogenase inhibition, multi-target immune modulation (dazdotuftide), and reactive aldehyde species inhibition (reproxalab). Additional searches addressed emerging cellular and immune-modulatory approaches, including regulatory T-cell–based therapies, T-cell co-stimulation blockade (abatacept), and B-cell–targeted therapies.

Reference lists of eligible publications were manually screened to identify additional relevant studies. Titles and abstracts were reviewed for relevance, followed by full-text assessment of potentially eligible articles. Only articles published in English were included. Case reports, case series, observational studies, and clinical trials were included when sufficient clinical detail regarding disease phenotype, therapeutic exposure, and treatment outcomes was available. Studies limited to preclinical or animal models were excluded. Infectious uveitis and scleritis were excluded during full-text review based on clinical diagnostic definitions. In total, 181 published articles reporting the clinical use of emerging therapeutic agents in NIU and NIS were identified and included in this review. This article is based exclusively on previously published studies and publicly available data and does not involve any new research with human participants or animals.

### 1.2 Emerging therapeutic agents and clinical evidence

To provide a structured overview of the therapeutic landscape, key characteristics of emerging agents are summarized in two tables. Table 1 outlines mechanisms of action, dosing strategies, and reported adverse events of emerging therapies in NIU and NIS. Table 2 summarizes the scope of clinical evidence, disease distribution across subtypes, and the certainty of evidence for clinical utility as assessed using the GRADE approach for each therapeutic class (6).

**TABLE 1:** Mechanisms of action, dosing, and reported adverse events of emerging therapies in non-infectious uveitis and scleritis.

Drugs	Type	Action	Route	Dosing commonly reported/used	FDA-approved indications	Adverse events reported in published ophthalmic studies
Anakinra	Biologic (recombinant, non-glycosylated human IL-1 receptor antagonist)	Blocks IL-1 receptor signaling (IL-1α/IL-1β)	SC, IV	SC: 100, 150 mg daily IV: 1–10 mg/kg/day	RA, CAPS including NOMID, DIRA	Injection-site reactions, local inflammatory skin nodules, injection-site discomfort, hypersensitivity reactions including anaphylaxis, hepatic enzyme elevation, GI symptoms, hypotension, isolated malignancy (papillary carcinoma of the urinary bladder), infections (upper respiratory tract infection, urinary tract infection, dental abscess)
Canakinumab	Biologic (fully human mAb)	Neutralizes IL-1β	SC, IV	SC: 150 mg every 6–8 weeks IV: 2 mg/kg monthly	CAPS including familial cold autoinflammatory syndrome and Muckle-Wells syndrome, systemic JIA, TRAPS, HIDS/MKD, FMF, active Still’s disease (including adult-onset Still’s disease), gout flares not responsive to NSAIDs/colchicine	
Gevokizumab	Biologic (recombinant humanized mAb)	Binds IL-1β at an allosteric epitope to dampen IL-1 signaling	SC, IV	SC: 30, 60 mg every 4 weeks IV: 0.3 mg/kg, 30, 60 mg IV every 4 weeks	Not FDA-approved for any indication	
Tocilizumab	Biologic (humanized mAb)	Blocks IL-6 receptor (both membrane-bound and soluble IL-6 receptors)	SC, IV	SC: 162 mg weekly IV: 4–8 mg/kg every 4 weeks	RA, GCA, polyarticular JIA, systemic JIA, CRS	Headache, fatigue, dizziness, nausea, GI upset, upper abdominal pain, sinus congestion, rhinorrhea, cough, arthralgia, acne vulgaris, alopecia, anxiety, sweating, flushing, injection-site reaction, infusion reaction, hypersensitivity reactions (drug eruption, diffuse urticaria, angioedema), neutropenia, leukopenia, thrombocytopenia, autoimmune thrombocytopenia, autoimmune anemia, liver enzyme elevation, hypertriglyceridemia, hypercholesterolemia, visual impairment, viral conjunctivitis, bullous impetigo, worsening uveitis, macular atrophy and ischemia, retinal detachment, phthisis bulbi, band keratopathy; infections (upper respiratory tract infection, bronchitis, pneumonia, pyelonephritis, urinary tract infection, cellulitis, sepsis, deep abscess, herpes zoster, COVID-19, diverticulitis)
Sarilumab	Biologic (fully humanized mAb)	Blocks IL-6 receptor (both membrane-bound and soluble IL-6 receptors)	SC	SC: 200 mg every 2 weeks	RA	
Vamikibart	Biologic (fully human mAb)	Neutralizes IL-6	Intravitreal	IVT: 0.25, 1 mg every 4 weeks for four initial doses then PRN	Not FDA-approved for any indication	Subconjunctival hemorrhage, increased intraocular pressure
Secukinumab	Biologic (fully human mAb)	Neutralizes IL-17A	SC, IV	SC: 150, 300 mg loading every 1–2 weeks for 2 times then every 2–4 weeks IV: 10 mg/kg every 2 weeks, or 30 mg/kg every 4 weeks	Plaque psoriasis, PsA, AS, non-radiographic axSpA, hidradenitis suppurativa	Headache, fatigue, upper abdominal pain, conjunctival hyperemia, eye pain, blurred vision, nasopharyngitis, hypokalemia, infections (urinary tract infection, pyelonephritis)
Ustekinumab	Biologic (humanized mAb)	Blocks IL-12/23 (p40 subunit)	SC, IV	SC: 45 mg at baseline, week 4 and every 12 weeks IV: 260 mg (up to 55 kg), 390 mg (55–85 kg), 520 (>85 kg) at baseline then 90 mg SC every 8 weeks	Plaque psoriasis, PsA, Crohn’s disease, UC	
Tofacitinib	Small molecule JAK inhibitor	Inhibits JAK1 and JAK3, with partial activity against JAK2	Oral	PO: 5,10 mg twice daily or 11 mg (extended- release) once daily	RA, PsA, UC, polyarticular JIA	Tachycardia, allergic conjunctivitis, keratitis, reduced visual acuity, floaters, ocular hyperemia, subretinal fluid, cystoid macular edema, iris adhesion, diarrhea, dyspepsia, fall, upper limb fracture, increased blood pressure, decreased neutrophil count, decreased white blood cell count, decreased vitamin D level, acne, phlebitis, infections (upper respiratory tract infection)
Baricitinib	Small molecule JAK inhibitor	Inhibits JAK1 and JAK2, with minimal activity against TYK2 and JAK3	Oral	PO: 4,5 mg once daily, 2 mg once daily (age < 6 years)	RA, alopecia areata, COVID-19 (hospitalized adults)	GI upset, nausea, fatigue, rash, acne, headache, cough, oropharyngeal pain, pyrexia, arthralgia, myalgia, ligament sprain, visual impairment, insomnia, worsening uveitis, retinal vascular occlusive events, infectious keratitis with corneal perforation, leukopenia, transaminitis, bladder prolapse, epilepsy, inflammatory bowel disease, spinal stenosis, suicidal ideation, infections (COVID-19, nasopharyngitis, upper respiratory tract infection, pneumonia, septic shock, urinary tract infection)
Upadacitinib	Small molecule JAK inhibitor	Inhibits JAK1	Oral	PO: 15, 30, 45 mg once daily	RA, PsA, AS, non-radiographic axSpA, atopic dermatitis, Crohn’s disease, UC	
Filgotinib	Small molecule JAK inhibitor	Inhibits JAK1	Oral	PO: 200 mg once daily	Not FDA-approved for any indication	
Brepocitinib	Small molecule JAK inhibitor	Inhibits JAK1 and TYK2	Oral	PO: 15, 45 mg once daily	Not FDA-approved for any indication	
KIO-100	Small molecule DHODH inhibitor	Selectively inhibits DHODH	Intravitreal	IVT: 0.3, 0.6, or 1.2 μg/0.1 mL	Not FDA-approved for any indication	
Dazdotuftide	Small molecule immunomodulator	Modulate innate immune responses (macrophage polarization hypothesis)	Topical	1% ophthalmic solution: four times daily	Not FDA-approved for any indication	Transient intraocular pressure elevation, conjunctival bleeding, conjunctival hyperemia
Reproxalap	Small molecule reactive aldehyde species inhibitor	Neutralizes reactive aldehydes generated during oxidative stress	Topical	0.5% ophthalmic solution: four times daily	Not FDA-approved for any indication	No published study
Abatacept	Biologic recombinant fusion protein	Blocks CD80/86-CD28 co-stimulation	SC, IV	SC: 125 mg weekly IV: 10 mg/kg (up to a maximum of 750 mg) at 0, 2, and 4 weeks, then every 4 weeks	RA, JIA, PsA	Eye irritation
Rituximab	Biologics (chimeric mAb)	Targeting the CD20 antigen	IV	IV: 1,000 mg on day 1 and 15, repeat after 6 months (rheumatologic protocol) or 375 mg/m^2^ body surface weekly for eight doses (foster protocol) or 375 mg/m^2^ body surface weekly for four doses with the ongoing maintenance therapy of 375 mg/m^2^ or 500 mg every 4–6 months (oncologic protocol)	NHL, CLL, pediatric mature B-cell malignancies, RA, GPA, MPA, PV	Skin reactions, allergic reaction, oral mycosis, diarrhea, fatigue, headache, post-infusion headache, weight gain, arthritis flare, branch retinal vein occlusion, rash
						Infusion-related reactions (shivering, flushing, urticaria, itching, anxiety, hypotension, palpitations, headache, ventricular tachycardia), allergic reactions requiring infusion cessation, conjunctivitis, ocular dryness, eyelid swelling, visual disturbances, increased intraocular pressure, dermatitis, peripheral neuropathy, fatigue, weight loss, mild arthralgia, leukopenia, cytopenias, hypogammaglobulinemia requiring intravenous immunoglobulin, infections (upper respiratory tract infection, sinusitis, conjunctivitis, urinary tract infection, bronchitis, bacterial pneumonia, septic shock, Pneumocystis jirovecii pneumonia, pulmonary fungal infection), viral infections (herpes zoster, ophthalmic zoster, herpetic oral ulcers)

AS, ankylosing spondylitis; axSpA, axial spondyloarthritis; CAPS, cryopyrin-associated periodic syndromes; COVID, coronavirus disease; CLL, chronic lymphocytic leukemia; CRS, cytokine release syndrome; DHODH, dihydroorotate dehydrogenase; DIRA, deficiency of interleukin-1 receptor antagonist; FDA, food and drug administration; FMF, Familial Mediterranean fever; GCA, giant cell arteritis; GI, gastrointestinal; GPA, granulomatosis with polyangiitis; HIDS/MKD, hyper-IgD syndrome/mevalonate kinase deficiency; IL, interleukin; IV, intravenous; IVT, intravitreal; JAK, janus kinase inhibitor; JIA, juvenile idiopathic arthritis; mAb, monoclonal antibody; MPA, microscopic polyangitis; NHL, non-Hodgkin lymphoma; NOMID, neonatal-onset multisystem inflammatory disease; NSAIDs, non-steroidal anti-inflammatory drugs; PDE4, phosphodiesterase-4; PKC, protein kinase C; PsA, psoriatic arthritis; PV, pemphigus vulgaris; RA, rheumatoid arthritis; SC, subcutaneous; TRAPs, tumor necrosis factor receptor–associated periodic syndrome; TYK2, tyrosine kinase 2; UC, ulcerative colitis.

**TABLE 2:** Summary of clinical evidence, disease distribution, and certainty of evidence (GRADE) for emerging therapeutic agents in non-infectious uveitis and scleritis.

Drug	Evidence of study	Diagnosis and/or underlying disease (*n*)				Certainty of evidence (GRADE)
		Anterior uveitis	Intermediate, posterior, panuveitis, and/or retinal vasculitis	Scleritis	Not specified	
IL-1 blockade						
Anakinra	Case report (9–15, 19), case series (21), retrospective study (8, 95), prospective study (22)	Behçet’s uveitis (1), CAPs (1)	Behçet’s uveitis (2), CINCA/NOMID syndrome (2), Blau syndrome (1), MS (1)	RA (3), RP (3), Behçet’s uveitis (1), PsA (1), Still’s disease (1), idiopathic (4), unspecified (3)	–	Moderate
	Multicenter, randomized controlled Trial (20)	–	Sarcoidosis (5), BSCR (3), Behçet’s uveitis (1), idiopathic (9)	–	–	–
Canakinumab	Case report (24, 25, 28, 30), case series (26, 27, 29)	Behçet’s uveitis (1), JIA (1), CINCA/NOMID syndrome (1)	Behçet’s uveitis (3), FMF (1), Blau syndrome (1), idiopathic (1)	–	–	Very low
Anakinra and canakinumab	Case series (16), retrospective study (17, 18, 282)	Behçet’s uveitis (7)	Behçet’s uveitis (46)	–	–	Low
Gevokizumab	Prospective, open-label pilot study (31)	–	Behçet’s uveitis (7)	–	–	Low
	Phase 1/2, open label, non-randomized, prospective, single-arm pilot trial (34)	–	–	RA (1), SLE and Sjögren’s syndrome (1), idiopathic (6)	–	–
	Prospective, open-label, randomized, parallel group, phase 2 multicenter trial (32) (randomized to different regimens of gevokizumab)	–	Behçet’s uveitis (21)	–	–	–
	Prospective, randomized, double-masked, placebo-controlled, event-driven phase 3 trial (33) (*N* = 83; 40 received gevokizumab, 43 received placebo)	–	Behçet’s uveitis (40)	–	–	–
IL-6 blockade						
Tocilizumab	Case report (40–43, 49, 53, 55, 56, 65, 69, 70, 74, 80, 84, 93, 94, 99, 100), case series (38, 39, 44, 50, 54, 63, 73, 78, 97, 98, 102, 103, 106)	JIA (10), scleroderma (1)	BSCR (10), Behçet’s uveitis (5), JIA (1), JIA with RVPT (1), RA (1), MCD (1), ROSAH (1), IRU (1), MS (1), RPC (1), pars planitis (1), idiopathic (8)	RP (5), VEXAS-RP (2), Takayasu arteritis (1), idiopathic (1),	JIA (2), Behçet’s uveitis (1)	Moderate
	Retrospective study (46, 47, 51, 57, 60–62, 64, 66–68, 72, 75–77, 83, 89, 92, 95, 206), multicenter retrospective study (45, 48, 52, 58, 59, 79, 81, 82, 85, 104, 105)	JIA (62), Behçet’s uveitis (6), RA (1), AS (1), idiopathic (1)	BSCR (61), Behçet’s uveitis (51), sarcoidosis (20), JIA (17), pars planitis (10), npAIR (8), VKH (5), SO (1), lupus nephritis (1), MFC (1), TINU (1), RA (1), idiopathic (85), not specified (13)	RA (4), IBD (1), idiopathic (1)	JIA (44), Behçet’s uveitis (11), sarcoidosis (7), AS (6), HLA-B27 (1), idiopathic (42), not specified (48)	–
IL-1 blockade						
	Prospective observational study (single-arm) (91)	–	Behçet’s uveitis (3)	–	–	–
	Multicenter, prospective, single-arm, phase 2 trial (APTITUDE) (88)	–	–	–	JIA (21)	–
	Multicenter, randomized, open-label, controlled, multicenter clinical (STOP-Uveitis) (86) (randomized to different regimens of tocilizumab)	–	Sarcoidosis (2), VKH (2), BSCR (2), PIC (1), Behçet’s uveitis (1), TINU (1), idiopathic (28)	–	–	–
	Multicenter, randomized controlled trial (20)	–	BSCR (11), sarcoidosis (8), Behçet’s uveitis (2), MFC (2), VKH (1), RPC (1), idiopathic (25)	–	–	–
Sarilumab	Phase 2, randomized, double-masked, placebo-controlled, parallel-group, multicenter study (SATURN) (107) (*N* = 58; 38 received sarilumab, 20 received placebo)	–	Not specified systemic conditions (13), idiopathic (25)	–	–	Moderate
Vamikibart	Phase 1, multipart, multicenter, non-randomized, open-label, multiple ascending dose study (DOVETAIL; NCT06771271)^**^	Not specified (11)	Not specified (26)	–	–	Moderate
	Phase 3, global, multicenter, randomized, double-masked, sham comparator-controlled trials (MEERKAT; NCT05642312) (*N* = 232; 152 received vamikibart, 80 received sham injection)^**^	–	–	–	–	–
	Phase 3, global, multicenter, randomized, double-masked, sham comparator-controlled trials (SANDCAT; NCT05642325) (*N* = 253; 171 received vamikibart, 82 received sham injection)^**^	–	–	–	–	–
IL-17 blockade						
Secukinumab	Phase 2 proof-of-concept trial (116)	Not specified (5)	Not specified (11)	–	–	Low
	Three multicenter, randomized, double-masked, placebo-controlled, dose-ranging phase 3 studies (117) (SHIELD, INSURE, and ENDURE) SHIELD (*N* = 118; 79 received secukinumab, 39 received placebo) INSURE (*N* = 31; 26 received secukinumab, 5 received placebo) ENDURE (*N* = 125; 91 received secukinumab, 34 received placebo)		Behçet’s uveitis (79), not specified (117)	–	–	–
	Multicenter, randomized, double-masked, dose-ranging, phase 2 clinical trial (118) (randomized to different regimens of secukinumab)	–	Not specified (37)	–	–	–
	Case report, (121–124), case series (119, 120)	AS (6), PsA (4),	PsA (1), tuberculosis (1), sarcoidosis (1)	–	–	–
IL-12/23 blockade						
Ustekinumab	Case report, (130, 131), case series (283)	Behçet’s uveitis, psoriasis and hidradenitis suppurativa (1), PsA (1)	Crohn,s disease (2)	–	–	Very low
	Prospective, non-randomized, uncontrolled, phase 2, two-arm pilot study (STAR; NCT02911116)^**^	–	Not specified (8)	–	–	–
JAK inhibitors						
Tofacitinib	Case report (101, 143, 145–148), case series, (142, 144, 149–151, 153, 165), retrospective study (95, 152), prospective cohort study (166)	AS (13), JIA (5), RA (4), PsA (1), Blau syndrome (1), idiopathic (3)	Sarcoidosis (4), VKH (2), JIA (1), AS (1), Blau syndrome (1), Behçet’s uveitis (1), MFC (1), SLE (1), axial spondyloarthritis (1), pars planitis (2), idiopathic (1)	RA (11), SLE (1), JIA (1), GPA (1), Sjögren’s syndrome (1), idiopathic (18)	–	Very low
Baricitinib	Case report (154), case series (144, 155), prospective cohort study (166)	JIA (22), idiopathic with positive ANA (4)	JIA (2), VKH (1), MFC (1), rheumatoid arthritis (1), ampiginous choroiditis (1), idiopathic (1)	RA (1)	–	Low
	Open-label, active-controlled, randomized, phase 3 clinical trial (JUVE-BRIGHT) (156) (*N* = 29; 24 received baricitinib, 5 received adalimumab)	JIA or positive ANA without JIA (24)	–	–	–	–
	Open-label, single-arm, Phase 3 clinical trial (JAKUVEITE; NCT05651880)*	–	–	–	–	–
Upadacitinib	Case report, (23, 158, 161, 163, 164), case series (159, 160, 162, 165), prospective cohort study (166)	JIA (3), AS (2), Behçet’s uveitis (1), idiopathic (1)	JIA (1), Behçet’s uveitis (2), AS (1), Crohn’s disease (1), BSCR (1), PIC (1), pars planitis (1), idiopathic (5)	RA (2), PsA (1), RA and UC with positive PR3-ANCA (1)	–	Very low
Filgotinib	Double-masked, placebo-controlled Phase 2 randomized clinical trial (HUMBOLDT) (167) (*N* = 72; 37 received filgotinib, 35 received placebo)	–	BSCR (5), sarcoidosis (5), RA (2), AS (1), VKH (1), idiopathic (21), not specified (2)	–	–	Moderate
Brepocitinib	Phase 2 randomized, double-masked, dose-ranging study (NEPTUNE; NCT05523765)*	–	–	–	–	Moderate
	Phase 3 randomized, double-masked, placebo-controlled study (CLARITY; NCT06431373)*	–	–	–	–	–
Dihydroorotate dehydrogenase						
KIO-100	Prospective, phase 1 open labeled, controlled clinical trial (185)		Serpiginous choroiditis (2), idiopathic (10)	–	–	Very low
Multi-target immune modulator						
Dazdotuftide (TRS01 eye drops)	Phase 3, prospective, multicenter, randomized, active-controlled, double-masked study (188) (*N* = 136; 87 received TRS01 1% eye drops, 49 received prednisolone acetate 1% eye drops)	Not specified (49)	–	–	–	Moderate
Reactive aldehyde species						
Reproxalap	Randomized, investigator-masked, comparator-controlled, parallel-group, multicenter phase 2 clinical trial (190) (*N* = 45; 15 received reproxalap 0.5%, 16 received reproxalap 0.5% and prednisolone 1%, 14 received prednisolone 1%)	Not specified (31)	–	–	–	Low
T-cell co-stimulation modulation						
Abatacept	Case report (195, 197, 198, 203, 204, 284), case series	JIA (4), idiopathic (1)	CTLA-4 deficiency (2), JIA (1), AZOOR (1), pars planitis (1), idiopathic (1)	–	PsA (1)	Very low
	Retrospective study (60, 92, 95, 206), multicenter retrospective study (200, 201, 285)	JIA (35)	JIA (2), idiopathic (1)	RA (3)	JIA (40)	–
	Two-center, prospective open label interventional proof-of-concept trial (202)	–	BSCR (15)	–	–	–
B cell targeted therapies						
Rituximab	Case report (211, 228, 253, 262, 264, 266, 267, 279), case series (211, 228, 253, 262, 264, 266, 267, 279)	JIA (1), type II essential cryoglobulinemia (1), not specified (1)	VKH (4), npAIR (4), CAR (3), SFU (2), BSCR (2), MS (2), MS with systemic sarcoidosis (1), Behçet’s uveitis (1), SLE (1), Waldenstrom’s macroglobulinemia (1)	GPA (14), RA (8), ANCA-associated (3), Sjogren’s syndrome (1), IgG4-related (1), surgical-induced (1), idiopathic (3)	–	Moderate
	Retrospective study (95, 212–214, 216, 217, 221, 223–226, 229, 239–242, 246, 250–252, 255, 257, 260, 269–271), multicenter retrospective study (215)	JIA (19), RA (1)	npAIR (34), VKH (30), GPA (9), MS (6), sarcoidosis (3), SLE (2), Behçet’s uveitis (1), SFU (1), idiopathic (8)	GPA (75), RA (7), ANCA-associated (5), RP (4), mixed connective tissue disorder (1), MPA (1), SLE (1), idiopathic (19)	JIA (8), MS (2), sarcoidosis (1)	–
	Randomized, single-blind, control study (pilot study) (219)	–	Behçet’s uveitis (10)	–	–	–
	Phase 1/2, prospective, non-randomized, open-label, single-center study (245)	–	npAIR (5)	–	–	–
	Prospective, dose-ranging, randomized, double-masked phase 1/2 clinical trial (278)	–	–	RA (4), GPA (1), systemic vasculitis (1), Cogan syndrome (1), idiopathic (5)	–	–

*Ongoing trial.

^**^Unpublished study. ANA, antinuclear antibody; ANCA, anti-neutrophil cytoplasmic antibody; AS, ankylosing spondylitis; AZOOR, acute zonal occult outer retinopathy; BSCR, birdshot chorioretinopathy; CAPs, cryopyrin-associated periodic syndromes; CAR, cancer-associated retinopathy; CINCA/NOMID, chronic infantile neurologic, cutaneous, and articular/neonatal-onset multisystem inflammatory disorder; CTLA, cytotoxic T-lymphocyte antigen; DHODH, dihydroorotate dehydrogenase; FMF, Familial Mediterranean fever; GPA, granulomatosis with polyangiitis; HLA: human leukocyte antigen; IBD, inflammatory bowel disease; Ig, immunoglobulin; IL, interleukin; IRU: immune recovery uveitis; JAK, janus kinase inhibitor; JIA, juvenile idiopathic arthritis; MCD, Multicentric Castleman disease; MFC, multifocal choroiditis; MPA, microscopic polyangitis; MS, multiple sclerosis; NIU, non-infectious uveitis; npAIR: non-paraneoplastic autoimmune retinopathy; PIC, punctate inner choroidopathy; PR3-ANCA, proteinase 3–specific anti-neutrophil cytoplasmic antibody; PsA, psoriatic arthritis; RA, rheumatoid arthritis; RASP, reactive aldehyde species; ROSAH: retinal dystrophy, optic nerve edema, splenomegaly, anhidrosis, and headache; RP, relapsing polychondritis; RPC: relentless placoid chorioretinitis; RVPT: retinal vasoproliferative tumor; SFU, subretinal fibrosis and uveitis syndrome; SLE, systemic lupus erythematosus; SO: sympathetic ophthalmia; TINU: tubulointerstitial nephritis and uveitis syndrome; TYK2, tyrosine kinase 2; UC, ulcerative colitis; VEXAS, vacuoles, E1 enzyme, X-linked, autoinflammatory, somatic syndrome; VKH, Vogt–Koyanagi–Harada disease.

## 2 Interleukin signaling pathway blockade

### 2.1 IL-1 blockade

Interleukin-1 blockade has demonstrated promising anti-inflammatory effects in experimental models of ocular inflammation and emerging clinical studies. IL-1, particularly IL-1β, is a key cytokine implicated in autoinflammatory and immune-mediated diseases and plays an important role in the pathogenesis of severe NIU, with elevated intraocular levels reported in inflammatory uveitic conditions, supporting a localized role of IL-1 signaling in ocular inflammation (7). Based on the available literature, three IL-1-targeted agents have been investigated in uveitis: anakinra, canakinumab, and gevokizumab. Anakinra and canakinumab are approved for several autoinflammatory conditions, including cryopyrin-associated periodic syndromes (CAPS), colchicine-resistant familial Mediterranean fever, TNF receptor-associated periodic syndrome, hyper-Immunoglobulin (Ig) D/mevalonate kinase deficiency, and Still’s disease, including systemic juvenile idiopathic arthritis (JIA). Anakinra is additionally FDA-approved for rheumatoid arthritis (RA) and deficiency of the IL-1 receptor antagonist.

#### 2.1.1 Anakinra

Anakinra (Kineret, Swedish Orphan Biovitrum AB, Stockholm, Sweden) is a recombinant, non-glycosylated human IL-1 receptor antagonist that competitively inhibits binding of both IL-1α and IL-1β to IL-1 receptor type 1. The earliest reports of anakinra use in uveitis, published in 2007, originated from pediatric autoinflammatory conditions, including a patient with Blau syndrome and severe steroid-refractory panuveitis, in whom systemic inflammation improved despite persistent ocular activity (8), and a child with chronic infantile neurologic, cutaneous, and articular (CINCA)/neonatal-onset multisystem inflammatory disorder (NOMID) with ocular involvement (9). Subsequent reports described NOMID/CAPS-associated uveitis and Still’s disease-related scleritis, with variable but often favorable responses to anakinra (10–12). More robust evidence for IL-1 blockade in NIU derives from off-label use in Behçet’s disease, supported by case reports, case series, and multicenter retrospective studies evaluating anakinra and canakinumab (7, 13–18). Across these studies, IL-1 inhibition was associated with rapid control of ocular inflammation, including panuveitis and retinal vasculitis, reduced uveitis relapse rates, and corticosteroid-sparing effects in patients refractory to conventional immunosuppressants and anti-TNF-α agents. Notably, a multicenter retrospective study of 30 patients with Behçet’s disease, including 16 with ocular involvement, emphasized the importance of treatment optimization strategies, such as dose escalation, shortening of dosing intervals, and intra-class switching, which were frequently associated with improved disease control. However, treatment discontinuation due to inefficacy or loss of response occurred in approximately one-quarter of patients (eight patients) (17). Selected reports also described successful use of anakinra in Behçet’s uveitis in the presence of latent tuberculosis infection, a setting in which anti-TNF-α therapy may be contraindicated (15). An additional case report described successful off-label use of anakinra in a patient with multiple sclerosis (MS)-associated panuveitis, leading to sustained improvement of VA, resolution of intraocular inflammation and macular edema, and long-term disease control without ocular or systemic adverse events (19). More recently, a multicenter, randomized controlled trial evaluated anakinra in patients with active, refractory non-anterior NIU. In this study, 112 patients across 27 centers were randomized (1:1:1) to receive adalimumab (*n* = 44), anakinra (*n* = 18; 100 mg daily), or tocilizumab (*n* = 50) over a 16-week treatment period. Interim analyses demonstrated a lack of efficacy in the anakinra arm, leading to early discontinuation (20). These findings suggest that the effectiveness of anakinra in broader uveitis populations may be limited despite encouraging results from observational studies in selected disease subsets.

In addition to uveitis, accumulating data support the use of anakinra in NIS. Initial case series reported improvement in RA-associated scleritis refractory to anti-TNF-α therapy (21). Subsequently, an open-label pilot study including 10 patients with severe and/or refractory non-necrotizing anterior or posterior scleritis, together with a retrospective cohort in which three patients received anakinra, demonstrated effective control of scleral inflammation, reduced relapse rates, sustained remission, and a substantial reduction in corticosteroid requirements (22, 23).

#### 2.1.2 Canakinumab

Canakinumab (Ilaris, Novartis Pharmaceuticals, Basel, Switzerland) is a fully human mAb selectively targeting IL-1β, providing sustained cytokine neutralization with a longer half-life and less frequent dosing compared with anakinra. Although larger retrospective cohort studies have evaluated IL-1 blockade in uveitis, these predominantly included patients treated with anakinra, with canakinumab used in a smaller subset, often as second-line or rescue therapy. Evidence specifically supporting canakinumab in uveitis therefore remains limited to case reports and small case series (24–30). Most of these reports involve off-label use in Behçet’s uveitis including patients switched from anakinra because of inefficacy or intolerance, with subsequent rapid control of ocular inflammation and stabilization of visual function (24, 26, 27). Isolated reports have also described favorable outcomes with canakinumab in uveitis associated with autoinflammatory diseases, such as familial Mediterranean fever, CINCA/NOMID syndrome, and Blau syndrome, as well as immune-mediated conditions including JIA-associated and idiopathic uveitis (25, 28–30). Overall, while canakinumab may offer mechanistic and pharmacokinetic advantages in selected patients, current evidence in uveitis remains observational and limited in scope.

#### 2.1.3 Gevokizumab

Gevokizumab (XOMA 052, XOMA Corporation, Berkeley, CA, United States) is a recombinant humanized mAb targeting IL-1β that modulates, rather than completely blocks IL-1 signaling by binding to an allosteric site on the IL-1 ligand and reducing its inflammatory activity. Evidence for gevokizumab in uveitis is derived primarily from clinical studies in Behçet’s disease-related uveitis. In an open-label pilot study involving seven patients with refractory posterior or panuveitis and/or retinal vasculitis, a single intravenous (IV) infusion of gevokizumab resulted in a rapid and sustained reduction in intraocular inflammation with improvement in VA (31). These findings were subsequently supported by a prospective, open-label, phase 2 multicenter trial enrolling 21 patients with Behçet’s uveitis, in which multiple IV and subcutaneous (SC) dosing regimens of gevokizumab were evaluated. Across treatment arms, gevokizumab was well-tolerated and effectively controlled acute uveitic exacerbations without requiring high-dose corticosteroids in addition to background immunosuppression (32). Despite encouraging signals from early-phase studies, the phase 3 EYEGUARD™ clinical development program ultimately did not meet its primary efficacy endpoints. In the randomized, double-masked, placebo-controlled EYEGUARD-B trial (NCT01965145), which included patients with Behçet’s disease-related posterior or panuveitis managed with background corticosteroid and immunosuppressive therapy, treatment with gevokizumab was not associated with a significant reduction in the incidence of ocular exacerbations or a prolongation of time to first flare when compared with placebo. However, secondary analyses suggested potential benefits, including preservation of VA, lower rates of macular edema and retinal vasculitis, and modest corticosteroid-sparing effects (33). Beyond uveitis, gevokizumab has also been evaluated in non-necrotizing anterior scleritis. In a phase 1/2 open-label pilot trial, gevokizumab was associated with a rapid reduction of scleral inflammation in seven of eight patients within 16 weeks (median, 2 weeks) after the first injection, with no significant changes in VA and no serious adverse events reported (34). Overall, available data suggest that gevokizumab may be beneficial in selected severe ocular inflammatory conditions; however, its clinical development remains limited, and the drug is not currently approved for any ocular indication.

#### 2.1.4 Safety considerations and summary

Across published uveitis studies, IL-1 inhibitors have demonstrated a favorable safety profile. The most frequently reported adverse events were injection-site reactions, particularly with anakinra, while serious adverse events were rare (17, 18, 20, 22). The largest safety dataset specific to uveitis derives from the randomized, double-masked, placebo-controlled EYEGUARD-B trial, in which 40 of 83 patients with Behçet’s uveitis received gevokizumab (33). In this study, gevokizumab was well-tolerated, with no safety concerns identified beyond standard-of-care corticosteroid and immunosuppressive therapy. Reported adverse events were largely attributable to the underlying disease or its complications. Drug hypersensitivity reactions were rare, occurring in a single patient and resolving within 1 day of injection, while skin reactions were infrequent and not considered treatment-related. No neutralizing anti-drug antibodies were detected, and IL-1β inhibition was not associated with an increased risk of opportunistic infections, including tuberculosis (33).

These observations are further supported by real-world safety data from a nationwide multicenter retrospective observational study of 475 patients treated with anakinra or canakinumab across a broad spectrum of on-label and off-label systemic inflammatory diseases (35). In this cohort, the overall risk of adverse events was 8.4% per patient-year, with approximately one-sixth of patients experiencing an adverse or serious adverse event during a mean follow-up exceeding 2 years. Most events were mild and cutaneous, while serious adverse events, mainly infections and rare anaphylactic reactions, were infrequent. Adverse event rates were not influenced by patient demographics, prior biologic exposure, or higher dosing regimens, and no tuberculosis reactivation was observed (35).

In summary, despite encouraging efficacy and safety signals in selected uveitis populations, particularly Behçet’s disease and autoinflammatory syndromes, the clinical use of IL-1 blockade in uveitis remains limited. The available evidence is largely confined to case reports, small case series, and retrospective studies, and randomized trial data have not demonstrated consistent efficacy in broader non-anterior uveitis populations. Practical considerations, including the need for parenteral administration, frequent dosing with agents such as anakinra, and limited long-term ocular-specific safety data, have further restricted widespread adoption in uveitis care.

### 2.2 IL-6 blockade

Interleukin-6 is a pleiotropic cytokine produced predominantly by monocytes, macrophages, T lymphocytes, and synovial fibroblasts, and plays a central role in regulating immune and inflammatory responses. In particular, IL-6 promotes the differentiation of CD4^+^ T helper (Th) cells into Th17 cells, which contribute to the pathogenesis of immune-mediated diseases, including NIU and NIS (2, 36). Elevated IL-6 levels have been consistently detected in the serum, aqueous humor, and vitreous of patients with multiple uveitis phenotypes, such as Vogt-Koyanagi-Harada (VKH) disease, Behçet disease, sarcoidosis, Fuchs heterochromic iridocyclitis, JIA-associated uveitis, and idiopathic uveitis (37). To date, direct inhibition of the IL-6 cytokine itself has not been established as an effective therapeutic strategy in uveitis. Instead, clinical experience has focused on blockade of the IL-6 receptor (IL-6R). Available biologics targeting this pathway include tocilizumab and sarilumab, with most clinical data in uveitis derived from studies of tocilizumab.

#### 2.2.1 Tocilizumab

Tocilizumab (Actemra/RoActemra^®^, Genentech/Roche, South San Francisco, CA, United States/Basel, Switzerland) is a humanized mAb that blocks both membrane-bound and soluble IL-6 receptors (mIL-6R and sIL-6R). Tocilizumab is FDA-approved for the treatment of RA, polyarticular and systemic JIA, giant cell arteritis, Chimeric Antigen Receptor (CAR) T-cell–induced cytokine release syndrome, severe Coronavirus disease (COVID)-19, and systemic sclerosis–associated interstitial lung disease. Although it is not approved for NIU or NIS, tocilizumab has been widely used off-label in immune-mediated ocular diseases, particularly in patients who are refractory to conventional immunosuppressive therapy (36).

In 2011, initial experience with tocilizumab in NIU was reported in two refractory cases: one with birdshot chorioretinopathy (BSCR) and one with idiopathic panuveitis, both with cystoid macular edema (CME) unresponsive to conventional therapy and anti-TNF agents. Monthly IV tocilizumab led to improved VA and reduced macular edema in both patients (38). Subsequent case reports, series, and retrospective studies have reported benefit of tocilizumab in NIU, including JIA-associated uveitis, BSCR, Behçet’s uveitis, HLA-B27-associated uveitis, sarcoidosis, pars planitis, retinal vasculitis, tubulointerstitial nephritis and uveitis, RA, MS, multicentric Castleman disease, retinal dystrophy–optic nerve edema–splenomegaly–anhidrosis–headache (ROSAH) syndrome, immune recovery uveitis, relentless placoid chorioretinitis, non-paraneoplastic autoimmune retinopathy (npAIR), scleroderma, autosomal dominant neovascular inflammatory vitreoretinopathy (ADNIV), and idiopathic uveitis (39–72). Additionally, several studies have reported favorable outcomes with tocilizumab for the treatment of macular edema related to NIU (73–84). In a retrospective study of 12 patients (16 eyes), mean central retinal thickness decreased from 516 μm at baseline to 274 μm at 12 months (*P* < 0.001) and remained stable through 24 months, with corresponding improvement in VA (77). However, not all eyes showed functional recovery; in some cases, VA failed to improve despite anatomical resolution of edema, likely reflecting irreversible, long-standing structural damage to the retina (78). A multicenter study of BSCR with CME showed that tocilizumab was significantly associated with reduced CME recurrence and lower daily corticosteroid use compared with conventional therapies and other biologic treatments (82). Focusing on tocilizumab versus anti-TNF-α agents, a multicenter retrospective study compared biologic therapies in patients with uveitic macular edema (UME) refractory to corticosteroids or conventional immunosuppressive drugs (81). A total of 204 patients were included (149 anti-TNF-α agents, 55 tocilizumab). At 6 months, response rates were 46.2% with anti-TNF-α agents and 58.5% with tocilizumab, with complete response in 21.8% and 35.8%, respectively. Tocilizumab was associated with a 2-fold higher odds of complete response, while relapse rates, risk of low vision, and corticosteroid-sparing effects were similar between groups (81). A 2023 multicenter retrospective study of 49 patients with refractory CME secondary to Behçet’s uveitis compared outcomes among adalimumab (*n* = 25), infliximab (*n* = 15), and tocilizumab (*n* = 9). Macular thickness decreased in all groups with resolution of CME by 1 year, and VA and intraocular inflammation improved similarly across treatments (85).

Evidence from prospective studies includes the STOP-Uveitis trial, a multicenter, randomized, open-label phase 1/2 pilot study evaluating IV tocilizumab (4 vs. 8 mg/kg every 4 weeks) in 37 adults with active non-infectious intermediate uveitis, posterior uveitis, or panuveitis (86). At the primary analysis at 6 months, approximately 43.5% of patients who had the potential for a 2-step decrease in vitreous haze achieved this outcome (40% in the 4 mg/kg group and 46.1% in the 8 mg/kg group), along with concurrent improvement in VA, reduction in CME, and decreased corticosteroid use. Differences between the two dose groups were not statistically significant, as the study was exploratory and not powered for dose comparison. A subsequent fluorescein angiography analysis of STOP-Uveitis demonstrated significant improvement in posterior segment inflammation at month 6, with reduced retinal vascular and disk leakage in both dose groups, no significant difference between the two groups, and approximately 83% of evaluable eyes showing improvement (87). Overall, STOP-Uveitis provides prospective evidence that tocilizumab can improve visual, anatomic, and inflammatory outcomes in non-anterior NIU, supporting IL-6 as a relevant therapeutic target while underscoring the need for larger controlled trials to confirm efficacy and define optimal dosing (86). Additional prospective evidence is provided by a multicenter, randomized controlled trial in patients with active, refractory non-anterior NIU. In this study, 112 patients across 27 centers were randomized (1:1:1) to receive adalimumab (*n* = 44), anakinra (*n* = 18), or tocilizumab (*n* = 50; 162 mg SC weekly). At the primary endpoint (week 16), no significant differences were observed between the tocilizumab and adalimumab group across inflammatory outcomes, including reduction in vitreous haze with concurrent corticosteroid tapering, as well as resolution of macular edema and retinal vasculitis (20).

In contrast to adult studies, prospective pediatric data remain limited. The APTITUDE study represents the largest prospective trial to date evaluating tocilizumab in children, and was designed as a multicenter, single-arm phase 2 study of SC tocilizumab in patients with JIA-associated uveitis refractory to both methotrexate and adalimumab. Tocilizumab was given at 162 mg every 3 weeks in children weighing <30 kg and every 2 weeks in those ≥30 kg. The primary endpoint was not achieved, as only 7 of 21 patients showed meaningful improvement in anterior chamber inflammation at 12 weeks. Despite this, benefit was observed in a subset of patients, particularly those with macular edema: 3 of 4 cases present at baseline showed resolution. Notably, all enrolled patients had highly refractory disease, and dosing followed standard regimens used for polyarticular JIA, without allowance for dose escalation (88). Following this prospective experience, retrospective data have provided additional support for SC tocilizumab in pediatric disease. In a subsequent retrospective study of 13 children with long-standing, chronic, and recalcitrant JIA-associated uveitis, SC tocilizumab (162 mg every 1 or 2 weeks) was associated with reduced flare rates and providing a steroid-sparing effect (89). More recent pediatric studies in 2025 further support the role of tocilizumab. A retrospective study of 15 children with NIU, IV or SC tocilizumab reduced relapse rates from 314 to 106 per 100 eyes/year, decreased systemic steroid use, and led to resolution of macular edema and retinal vasculitis with stable VA (90). Another pediatric study focused on retinal vasculitis showed that tocilizumab significantly improved retinal vasculitis and ocular inflammation, with efficacy comparable to adalimumab and infliximab, supporting its use particularly after anti-TNF agent failure (72).

Not all studies have shown favorable outcomes with tocilizumab in NIU. A small prospective, single-arm observational study evaluated IV tocilizumab (8 mg/kg every 4 weeks) for acute attacks of Behçet’s uveitis in three patients with panuveitis refractory to prior biologics, including anti-TNF-α agents and interferon-α2a. Although transient improvement was observed after the first 1–4 infusions, all three patients experienced recurrent uveitis attacks, leading to early termination of the study (91). Similarly, in a long-term cohort of 125 patients with JIA-associated uveitis followed for a mean of 9.2 years at a national referral center, seven patients received tocilizumab, with three discontinuing therapy because of loss of efficacy (92).

Clinical experience with tocilizumab in NIS has been mainly derived from case reports and small case series, largely involving patients with severe, refractory disease who had failed conventional immunosuppression and anti-TNF-α therapy. Across these reports, IV or SC tocilizumab (typically 8 mg/kg every 4 weeks or 162 mg weekly) was associated with rapid control of scleral inflammation, resolution of necrosis in necrotizing scleritis, and sustained remission in many cases. Tocilizumab has shown particular benefit in scleritis associated with systemic inflammatory conditions such as relapsing polychondritis, giant cell arteritis, Takayasu arteritis, and VEXAS syndrome, often allowing significant corticosteroid tapering or discontinuation (47, 93–101).

Subcutaneous tocilizumab is increasingly used in clinical practice to reduce hospital visits and facilitate home-based treatment, leading some stable patients to switch from IV to SC administration. The efficacy of different routes of tocilizumab administration has shown variable results. Small case series suggest that some patients relapse after switching from IV to SC tocilizumab, particularly those with active retinal vascular leakage before the switch, whereas patients with minimal baseline leakage may remain stable on SC therapy (102, 103). Alternatively, improvement in central foveal thickness, ocular inflammation, and VA after switching from IV to SC tocilizumab has been reported in an isolated case of JIA-associated UME (80). Larger retrospective data published in 2025 from two multicenter retrospective studies showed that IV and SC tocilizumab were similarly effective in controlling intraocular inflammation, although one study suggested faster resolution of UME with IV dosing (104, 105). Importantly, failure of SC therapy does not preclude benefit from IV re-challenge. A 2023 case series reported 4 patients with chronic NIU who improved after switching back from SC to IV tocilizumab, with rapid clinical response, steroid tapering in most cases, and no major safety concerns (106).

#### 2.2.2 Sarilumab

Sarilumab (Kevzara, Regeneron Pharmaceuticals/Sanofi, Tarrytown, NY, United States/Paris, France) is a fully human mAb directed against the alpha subunit of the IL-6R, binding both membrane-bound and soluble forms of the receptor. Sarilumab is FDA-approved for moderately to severely active RA, polymyalgia rheumatica, and, in patients ≥63 kg, polyarticular JIA.

Clinical evidence for sarilumab in uveitis is limited to a single prospective trial, the SATURN study. This phase 2, randomized, double-masked, placebo-controlled multicenter trial evaluated SC sarilumab 200 mg every 2 weeks in 58 patients with non-infectious intermediate, posterior, or panuveitis (107). At week 16, the primary composite endpoint defined as either a ≥ 2-step reduction in vitreous haze or reduction of systemic corticosteroids to <10 mg/day was achieved in 46.1% of sarilumab-treated patients compared with 30.0% of placebo-treated patients based on central reading center assessment using color fundus photographs (*P* = 0.235), and in 64.0% versus 35.0%, respectively, based on investigator assessment (*P* = 0.037). Sarilumab was also associated with greater improvement in VA and greater reduction in central subfield thickness, particularly among eyes with baseline macular edema (107).

#### 2.2.3 Vamikibart

Vamikibart (Genentech, South San Francisco, CA, United States) is a recombinant, fully human IgG2 mAb designed for intravitreal (IVT) administration. The molecule selectively binds IL-6. Vamikibart is intended to suppress downstream inflammatory signaling while minimizing systemic exposure. In the first-in-human, open-label Phase 1 DOVETAIL study (NCT06771271), IVT vamikibart was evaluated in patients with UME (108). Treatment was associated with rapid reductions in central retinal thickness and concurrent improvements in best-corrected visual acuity (BCVA) (109). Subsequently, two parallel, randomized, double-masked, sham-controlled Phase 3 trials, MEERKAT (NCT05642312) and SANDCAT (NCT05642325), were conducted in patients with macular edema secondary to NIU (110, 111). In both studies, participants received IVT vamikibart (0.25 or 1 mg) or sham every 4 weeks for four initial doses, followed by as-needed dosing. The primary endpoint was the proportion of patients achieving a ≥ 15-letter gain in BCVA at week 16, with key secondary endpoints including mean change from baseline in BCVA and central subfield thickness (CST).

In MEERKAT, both dose groups met the primary endpoint, with absolute differences versus sham of 19.9% (*P* = 0.0008) for the 0.25 mg group and 36.9% (*P* < 0.0001) for the 1 mg group. Mean BCVA gains were +9.6 letters (*P* = 0.0002) and +12.8 letters (*P* < 0.0001), compared with +3.5 letters in the sham arm, and corresponding reductions in CST were −187.5 μm and −196.1 μm (both *P* < 0.0001). In SANDCAT, numerically greater proportions of patients achieved ≥15-letter gains with vamikibart compared with sham (20.7% difference for the 0.25 mg group; 10.9% for the 1 mg group), although statistical significance for the primary endpoint was not consistently demonstrated. Improvements in mean BCVA and CST were also observed in the active-treatment arms (112). Across both Phase 3 trials, IVT vamikibart demonstrated an overall favorable tolerability profile. Treatment-related ocular adverse events and intraocular inflammation were infrequent, and no cases of retinal occlusive vasculitis were reported. The most frequently observed ocular adverse events included conjunctival hemorrhage and increased intraocular pressure (112).

#### 2.2.4 Safety considerations and summary

Across published uveitis reports, tocilizumab has generally been well-tolerated. Reported adverse events mirror established rheumatology safety signals and most commonly include infusion or hypersensitivity reactions, gastrointestinal symptoms, and laboratory abnormalities such as neutropenia, elevated liver enzymes, and dyslipidemia (20, 40, 43, 45, 47, 48, 51, 52, 58, 64, 68, 71, 72, 80, 85, 92, 97). Serious adverse events are uncommon and are mainly infectious, including pneumonia, herpes zoster, sepsis from cellulitis, pyelonephritis, and diverticulitis. Tuberculosis has not been reported, although most patients underwent pre-treatment screening (20, 48, 58, 82, 85, 98).

In a large multicenter retrospective study of UME, adverse events occurred in 12.7% of TCZ-treated patients, including abscess, pneumonia, injection-site reaction, bronchitis, and pyelonephritis (81). In the STOP-Uveitis trial, most adverse events were mild; two cases of neutropenia occurred in the IV 8 mg/kg group, and no new major safety concerns were identified (86). In the pediatric APTITUDE trial, no serious adverse events were observed; the most frequent events were injection-site reactions, arthralgia, and headache (88). For sarilumab, uveitis-specific safety data are limited to the phase 2 SATURN trial, in which ocular and systemic adverse events were similar between sarilumab and placebo. Neutropenia developed in 7.9% of sarilumab-treated patients, which was reversible (107).

Overall, evidence for IL-6 blockade in NIU is dominated by case reports/series and retrospective cohorts, with some prospective and controlled data. Most reports involve JIA-associated uveitis, BSCR, Behçet’s uveitis, sarcoidosis, and idiopathic uveitis, often showing controlled ocular inflammation, fewer relapses, and steroid-sparing effects, though non-response and loss of efficacy occur. A particularly notable finding is the accumulating evidence for favorable outcomes in the treatment of macular edema in NIU. Future needs include adequately powered randomized trials, head-to-head comparisons with anti–TNF-α agents, and clarification of the optimal route and dosing strategy.

### 2.3 IL-17 blockade

Dysregulated Th17 immune responses contribute significantly to the pathogenesis of NIU. Within the IL-23/IL-17 axis, IL-23 functions upstream to drive and sustain pathogenic Th17 responses, whereas IL-17 mediates downstream tissue inflammation. In addition, IL-23 promotes IL-17 production from both adaptive and innate immune cells, linking innate immune activation to chronic ocular inflammation (113). Increased IL-23/IL-17A signaling has been demonstrated in Behçet’s disease and VKH disease, supporting the IL-17 axis as a biologically plausible therapeutic target (114, 115). Clinical experience with IL-17-targeted biologics in ocular inflammation is currently limited to secukinumab in NIU, with heterogeneous outcomes and no available evidence in NIS.

#### 2.3.1 Secukinumab

Secukinumab (Cosentyx, Novartis Pharmaceuticals, Basel, Switzerland) is a fully human mAb that selectively inhibits IL-17A, thereby attenuating downstream inflammatory signaling and immune cell activation. The agent is approved by the FDA for the treatment of plaque psoriasis, psoriatic arthritis (PsA), ankylosing spondylitis (AS), non-radiographic axial spondyloarthritis, enthesitis-related arthritis, and hidradenitis suppurativa. Secukinumab was initially evaluated as an IV formulation in a phase 2 proof-of-concept study involving patients with active chronic NIU, demonstrating rapid reductions in ocular inflammation with favorable tolerability.(116) Subsequently, SC secukinumab was evaluated in three multicenter, randomized, double-masked, placebo-controlled, dose-ranging phase 3 trials (SHIELD, INSURE, and ENDURE) enrolling patients with intermediate, posterior, and panuveitis (117). These studies examined distinct clinical settings, including Behçet’s disease-associated active posterior uveitis or panuveitis (SHIELD), active non-Behçet’s NIU requiring immunosuppression (INSURE), and quiescent NIU receiving maintenance therapy (ENDURE). Across all trials, secukinumab did not significantly reduce uveitis recurrence compared with placebo, resulting in early termination of the INSURE and ENDURE studies after prespecified interim analyses. Nevertheless, secondary outcomes from SHIELD and INSURE suggested a possible reduction in concomitant immunosuppressive medication use. However, these findings should be interpreted cautiously given the overall negative primary efficacy results and early study termination (117). A subsequent multicenter, randomized, double-masked, dose-ranging phase 2 trial directly compared SC and IV secukinumab in patients with active non-infectious intermediate uveitis, posterior uveitis, or panuveitis requiring corticosteroid-sparing therapy (118). IV secukinumab demonstrated significantly higher responder rates than SC administration, with faster onset of response and numerically greater improvements in remission rates, vitreous haze, and VA outcomes. Pharmacokinetic analyses revealed substantially higher and more sustained serum drug concentrations with IV dosing, suggesting that standard SC regimens may not achieve therapeutic exposure in ocular inflammation. Both IV and SC formulations were generally well-tolerated, although the IV formulation is not widely available for routine clinical use (118).

Given the conflicting efficacy outcomes observed in controlled clinical trials, subsequent evidence for secukinumab in NIU has largely been limited to case reports and small case series. Most published experiences describe secukinumab use as second- or third-line therapy in patients with uveitis refractory to anti-TNF-α agents (119, 120), or in complex clinical settings, including uveitis developing during anti–TNF-α therapy (121), anti-TNF-associated tuberculosis-related uveitis (122), new-onset spondyloarthropathies in patients with prior uveitis (123), or anti-TNF-induced sarcoidosis (124). Real-world outcomes have been heterogeneous. Small case series in HLA-B27-associated anterior uveitis and spondyloarthropathy-spectrum disease have reported both meaningful reduction in uveitis recurrences with good tolerability and, conversely, persistent ocular flares with inadequate systemic disease control (119, 120). Individual case reports further describe variable responses following a switch from anti-TNF-α therapy, including improvement in ocular inflammation and systemic manifestations in selected patients, as well as successful use in complex scenarios such as anti-TNF-induced sarcoidosis (122, 124). Collectively, these observations suggest that secukinumab may have a role in carefully selected patients but underscore the variability of response and the lack of robust evidence supporting its routine use in NIU. Additionally, clinical data suggest worse control of uveitis compared to adalimumab. A large nationwide real-world cohort comparing multiple biologic disease-modifying antirheumatic drugs, secukinumab was associated with a higher risk of incident acute anterior uveitis compared with adalimumab (adjusted hazard ratio 2.17, 95% CI 1.27–3.71) (125). A *post hoc* pooled analysis of 11 placebo-controlled phase 3 trials reported an incidence of uveitis in patients with axial spondyloarthritis and PsA treated with secukinumab during the placebo-controlled phase. In axial spondyloarthritis, the exposure-adjusted incidence rate was numerically lower with secukinumab than with placebo (1.29 vs 1.72 per 100 patient-years), while in PsA, uveitis incidence remained low (0.71 per 100 patient-years with secukinumab 300 mg), with no events reported in the secukinumab 150 mg or placebo groups (126).

### 2.3.2 Safety considerations and summary

Overall, IL-17A inhibition with secukinumab has demonstrated an acceptable safety profile in NIU, with no consistent increase in ocular serious adverse events and predominantly mild-to-moderate systemic toxicity across clinical trials and real-world reports. Higher systemic exposure achieved with IV dosing was not associated with increased serious adverse events, suggesting that safety is unlikely to be the primary barrier to efficacy.

From a future perspective, other IL-17–targeted mAb with established efficacy in spondyloarthropathies, including ixekizumab (IL-17A) and brodalumab (IL-17RA), warrant further investigation in ocular inflammatory disease. Bimekizumab, which concurrently neutralizes both IL-17A and IL-17F, is another agent of interest given its broader inhibition of the IL-17 pathway; however, clinical evidence in NIU and NIS remains limited. In pooled analyses from phase 2b/3 trials conducted in rheumatologic populations, bimekizumab was associated with a lower incidence of acute anterior uveitis compared with placebo during the double-blind treatment period (127). The exposure-adjusted incidence rate of uveitis was numerically lower in bimekizumab-treated patients, including those with a prior history of uveitis, and remained low with longer-term exposure across pooled phase 2b/3 and open-label extension data (127). These findings should be interpreted cautiously given the low number of events and the absence of formal ophthalmologic adjudication. Experimental autoimmune uveitis models provide a potential mechanistic framework, suggesting that selective IL-17A blockade may allow persistent Th17 pathogenicity through compensatory upregulation of IL-17F and GM-CSF, whereas IL-17F inhibition does not appear to induce a similar response (128). However, whether these mechanistic observations translate into clinically meaningful differences in ocular inflammatory disease remains to be determined.

### 2.4 IL-12/23 blockade

Among biologic agents targeting upstream regulators of the IL-23/IL-17 axis, ustekinumab (Stelara, Janssen Biotech, Horsham, PA, United States) is a human mAb directed against the shared p40 subunit of IL-12 and IL-23. The agent has demonstrated a well-established safety and efficacy profile in psoriasis and PsA and is approved for the treatment of plaque psoriasis, PsA, Crohn’s disease, and ulcerative colitis. The biological rationale for ustekinumab in uveitis is supported by evidence implicating both Th1 and Th17 pathways, including elevated serum IL-12 and IL-23 levels and correlations between IL-23 and ocular inflammatory activity. Through blockade of the p40 subunit, ustekinumab suppresses IL-12 activity in T cells and natural killer cells, as well as IL-23 production by dendritic cells and macrophages, thereby targeting both adaptive and innate immune responses (129).

Clinical evidence for ustekinumab in NIU has primarily emerged from isolated case reports and small case series involving patients with multisystem immune-mediated disease who were refractory or intolerant to anti-TNF-α therapy. One report described successful treatment with ustekinumab in a patient with Behçet’s disease and anterior uveitis concomitant with psoriasis and hidradenitis suppurativa, with immunologic assessment demonstrating post-treatment downregulation of IL-12– and IL-23–associated pathways (130). Another case involved severe pustular psoriasis and PsA complicated by anterior uveitis, refractory to cyclosporine and intolerant to anti-TNF-α therapy. Ustekinumab led to resolution of uveitis with sustained remission of both ocular and systemic manifestations (131). Additional evidence comes from a small case series of two patients with Crohn’s disease-associated NIU, both experiencing recurrent, sight-threatening flares despite anti-TNF-α therapy. In these cases, ustekinumab induction followed by SC maintenance was associated with sustained suppression of uveitis activity over several months, without reported recurrences (23). The most systematic evaluation of ustekinumab in NIU derives from the STAR study (Ustekinumab for the Treatment of Active Sight-Threatening Uveitis; NCT02911116), a prospective, non-randomized, uncontrolled phase 2 pilot trial. The study enrolled patients with active intermediate uveitis, posterior uveitis, or panuveitis and evaluated 2 dosing strategies: SC administration alone or IV induction followed by SC maintenance. Of the eight enrolled patients, six completed the week 16 assessment and were included in the final analysis. All evaluable patients demonstrated a treatment response, defined by the absence of active inflammatory chorioretinal lesions and/or absent or reduced retinal vascular leakage, anterior chamber cell grade ≤ 0.5+, and vitreous haze grade ≤ 0.5+. No serious adverse events were reported (132). To date, the results of the STAR study have not been published in a peer-reviewed journal, and available data remain limited to registry-reported outcomes.

*Post hoc* analyses from large phase 3 Crohn’s disease trials (UNITI-1, UNITI-2, and IM-UNITI) showed that uveitis was uncommon among patients treated with ustekinumab, and only a small number of new-onset uveitis cases were reported during therapy. Among 941 patients who received ustekinumab during induction therapy, 23 (2.4%) had baseline iritis or uveitis, of whom 15 experienced resolution by week 6 (*p* = 0.019). In the IM-UNITI maintenance analysis, 8 of 263 patients (3.0%) had baseline iritis or uveitis, with resolution observed in 7 patients by Week 52 (*p* = 0.070). Overall, these data indicate that evidence supporting ustekinumab for ocular extraintestinal manifestations of inflammatory bowel disease remains limited (133). Additional epidemiologic data are derived from large observational studies in systemic inflammatory diseases. In a nationwide cohort of patients with moderate-to-severe psoriasis, ustekinumab was associated with a significantly lower incidence of uveitis compared with anti-TNF-α agents, with incidence rates of 2.34 versus 10.16 per 1,000 person-years, respectively (adjusted hazard ratio 0.22, 95% CI 0.11–0.41).(134)

## 3 Intracellular signaling and metabolic pathway modulators

### 3.1 Janus kinase (JAK) inhibitors

Janus kinase inhibitors target a family of intracellular tyrosine kinases that mediate signaling for multiple cytokines involved in immune regulation and hematopoiesis. Following cytokine binding to cell-surface receptors, receptor-associated JAKs become activated and phosphorylate signal transducer and activator of transcription (STAT) proteins. Phosphorylated STATs subsequently dimerize and translocate to the nucleus, where they regulate transcription of immune-related genes, thereby linking extracellular cytokine signaling to downstream genomic responses (135).

The JAK family comprises four members, JAK1, JAK2, JAK3, and tyrosine kinase 2 (TYK2), which associate with specific cytokine receptors to form distinct yet partially overlapping signaling complexes (136). Notably, several cytokines implicated in the pathogenesis of ocular inflammation, including IL-2 and IL-6, signal through the JAK–STAT pathway. As a result, pharmacologic inhibition of JAK activity offers a means of simultaneously modulating multiple inflammatory pathways using orally administered small-molecule agents that competitively bind the ATP-binding domain of JAKs (137).

The JAK–STAT pathway has rapidly become a therapeutic target across multiple immune-mediated diseases, including myeloproliferative neoplasms, RA and related arthropathies, inflammatory dermatologic disorders, and inflammatory bowel disease (138). Unlike biologic therapies, which require IV or SC administration, JAK inhibitors are orally administered and demonstrate relatively rapid onset of action. Despite increasing clinical adoption, their application in NIU remains investigational, with use off-label supported by a growing but still limited evidence base (137).

To date, five JAK inhibitors, tofacitinib, baricitinib, upadacitinib, filgotinib, and brepocitinib, have been evaluated in clinical studies of NIU. Among these agents, only filgotinib and brepocitinib have been assessed in randomized, controlled clinical trials for NIU. In contrast, evidence supporting the use of tofacitinib and upadacitinib is derived primarily from case reports, case series, and observational cohorts. Nevertheless, when viewed collectively, available data suggest that JAK inhibition may represent a therapeutic option for patients with uveitis refractory to conventional systemic immunosuppression or corticosteroid-based strategies (139). In parallel, interest has extended to TYK2-selective inhibition targeting type I interferon and IL-12/IL-23 pathways; however, the Phase 2 OPTYK-1 trial evaluating the oral TYK2 inhibitor ESK-001 in NIU was discontinued in late 2024 due to insufficient efficacy (140).

#### 3.1.1 Tofacitinib

Tofacitinib (Xeljanz, Pfizer Inc., New York, NY, United States) is the first FDA-approved JAK inhibitor and preferentially inhibits JAK1 and JAK3, with partial activity against JAK2 (141). It is approved for RA, PsA, ulcerative colitis, and polyarticular JIA. In ocular inflammation, early case series reported meaningful and sustained control of non-infectious anterior and intermediate uveitis, and scleritis in patients who had failed multiple steroid-sparing therapies (142). Subsequent case reports demonstrated benefit in JIA–associated anterior uveitis with refractory macular edema, as well as in chronic bilateral NIU resistant to multiple conventional immunosuppressive agents and adalimumab (143–145). Additional reports have also described the successful use of tofacitinib in patients with VKH disease refractory to standard immunomodulatory therapy, resulting in improved VA and resolution of subretinal fluid (146). More recently, a case of refractory panuveitis with coexisting non-radiographic axial spondyloarthritis demonstrated sustained disease control with tofacitinib (10 mg/day) following failure of both TNF-α and IL-17 inhibitors. After an initial induction phase with occasional early recurrences, the patient remained free of ocular inflammatory attacks for 3 years, with stable VA and no reported adverse events (147). Clinical improvement has also been documented in scleritis, including resolution of necrotizing disease refractory to mycophenolate mofetil and systemic corticosteroids, and in several case series reporting complete or near-complete remission of NIS following failure or intolerance of conventional immunomodulatory therapy (23, 148–151). A pediatric case involving a 13-year-old boy with NIS reported sustained remission following a cost-related switch from tocilizumab to tofacitinib (23). In addition, one of the larger retrospective cohorts to date, including 35 patients with non-infectious ocular inflammation, reported a favorable inflammatory response in 94% of cases, providing important real-world evidence for its potential therapeutic role (152). Pediatric data further support this trend, with most children achieving remission in a recent case series of treatment-resistant NIU or NIS treated with tofacitinib (153).

#### 3.1.2 Baricitinib

Baricitinib (Olumiant, Eli Lilly and Company, Indianapolis, IN, United States) is a selective JAK1 and JAK2 inhibitor with minimal activity against TYK2 and JAK3 and is approved for the treatment of moderately to severely active RA in multiple regions worldwide. In a small case series, all three patients with recalcitrant JIA-associated uveitis treated with baricitinib demonstrated improvement in ocular inflammation, although articular disease response was limited (144). A separate case report described baricitinib use in a patient with panuveitis and coexisting seronegative RA, resulting in improvement of systemic disease activity and resolution of anterior chamber inflammation, corneal infiltrates, vitreous opacity, and serous retinal detachment (154). In a recent three-patient case series, baricitinib induced sustained remission for over 1 year in two patients with refractory isolated NIU, while the third patient required a switch to upadacitinib to achieve improved disease control (155). More recently, the Phase 3 JUVE-BRIGHT trial evaluated baricitinib against adalimumab in 29 pediatric patients who received treatment (24 baricitinib, 5 adalimumab) for JIA-associated or ANA-positive anterior uveitis. The study did not meet its primary efficacy endpoint, likely reflecting recruitment challenges and limited statistical power inherent to this rare pediatric population (156). The ongoing JAKUVEITE trial aims to further assess baricitinib in non-infectious non-anterior uveitis refractory to prior biologic therapy (157).

#### 3.1.3 Upadacitinib

Upadacitinib (Rinvoq, AbbVie Inc., North Chicago, IL, United States) is a selective JAK1 inhibitor approved for multiple immune-mediated diseases, including RA, PsA, AS, atopic dermatitis, and inflammatory bowel disease. Its selective inhibition of JAK1, with relative sparing of JAK2 signaling, may mitigate hematologic adverse effects (136). The first reported ocular application demonstrated remission of JIA-associated anterior uveitis refractory to multiple conventional and biologic agents, including tofacitinib (158). Subsequent case series have reported benefit in diverse uveitic entities, including Behçet’s disease, Crohn’s disease-associated uveitis, and BSCR, with improvements in VA, intraocular inflammation, macular edema, and choroidal involvement (159, 160). Pediatric evidence also suggests efficacy of upadacitinib in refractory uveitis, with reports of rapid improvement in idiopathic chronic panuveitis and durable remission in JIA-associated anterior uveitis (161, 162). In addition, a recent case report described refractory scleritis associated with RA and ulcerative colitis, complicated by PR3-ANCA positivity, in which disease control was not sustained with tofacitinib but was achieved after switching to upadacitinib, allowing durable remission and withdrawal of systemic corticosteroids and methotrexate (163). More recently, a case report described necrotizing scleritis refractory to multiple biologic agents, including certolizumab, etanercept, and tocilizumab, that was successfully treated with upadacitinib (15 mg/day), with complete resolution of ocular pain and inflammation, regression of nodular lesions, and stabilization of scleral thinning within 2 months, along with successful corticosteroid tapering and sustained remission at 6 months without reported adverse events (164). Additional series have also noted reduced flare frequency in NIU and NIS following initiation of tofacitinib or upadacitinib (165). Consistently, registry-based data from the AutoInflammatory Disease Alliance (AIDA) suggest potential effectiveness across uveitis and scleritis, although conclusions are limited by observational design and sample size (166).

#### 3.1.4 Filgotinib

Filgotinib (Jyseleca, Galapagos NV/Gilead Sciences, Mechelen, Belgium/Foster City, CA, United States) is a selective JAK1 inhibitor approved in Europe, the United Kingdom, and Japan for RA and ulcerative colitis. By preferentially inhibiting JAK1-mediated signaling, it modulates cytokines such as IL-6 and type I interferons implicated in NIU pathogenesis. The Phase 2 HUMBOLDT trial demonstrated that filgotinib significantly reduced treatment failure in non-infectious intermediate, posterior, and panuveitis compared with placebo, along with improvements in anterior chamber inflammation, VA, and retinal thickness (167). Although the study was terminated prematurely for non-scientific reasons, filgotinib showed an acceptable safety profile and these results provide proof-of-concept randomized evidence supporting JAK inhibition in NIU. Notably, despite regulatory approval for RA in Japan and the European Union, further development of filgotinib for uveitis, including in the United States, was discontinued for non-scientific reasons.

#### 3.1.5 Brepocitinib

Brepocitinib (PF-06700841, Pfizer Inc., New York, NY, United States) is a dual JAK1/TYK2 inhibitor designed to provide broader immunomodulation while minimizing cytopenias and immunodeficiency associated with JAK2 and JAK3 inhibition (139). In the Phase 2 NEPTUNE trial evaluating brepocitinib in non-anterior NIU, brepocitinib demonstrated dose-dependent efficacy, with lower treatment failure rates observed in the higher-dose group (45 vs. 15 mg once daily) (168, 169). The ongoing Phase 3 CLARITY trial aims to further evaluate its safety and efficacy in a larger global population with active non-infectious intermediate, posterior, or panuveitis (170).

#### 3.1.6 Safety considerations and summary

Janus kinase inhibitors are associated with infectious complications, particularly herpes zoster, as well as the risk of thromboembolic events, malignancies, and gastrointestinal perforation, primarily based on data from tofacitinib and baricitinib (171–176). Meta-analyses of randomized trials have indicated a possible increase in venous thromboembolism risk with JAK inhibitors compared with placebo or anti-TNF agents, although findings have been inconsistent; higher doses of tofacitinib have been associated with an increased risk of pulmonary embolism (176). Additional adverse effects include upper respiratory and urinary tract infections and hyperlipidemia (141), with a possible increase in major adverse cardiovascular events, especially in older patients and those with pre-existing cardiovascular disease (165, 177). Filgotinib has been associated with transient, dose-dependent reductions in semen parameters without sustained hormonal effects (178), contributing to regulatory concerns despite approvals outside the U.S. Although the HUMBOLDT trial was terminated early for business reasons, a higher rate of drug-related adverse events, including visual disturbance, insomnia, urinary tract infection, and gastrointestinal symptoms, was observed with filgotinib compared with placebo (167). In contrast, available data from the NEPTUNE trial have not identified major systemic adverse events with brepocitinib to date (169). Regarding ocular adverse events, accumulating evidence suggests that JAK inhibitors may be associated with cytomegalovirus retinitis. Four cases have been reported in patients receiving tofacitinib (2 with monotherapy and 2 with concomitant methotrexate and corticosteroids), as well as 1 case with upadacitinib monotherapy (179–182).

In summary, JAK inhibitors remain in an early phase of clinical integration for NIU. While current evidence is largely derived from refractory populations and non-randomized studies, ongoing Phase 3 trials are expected to provide more definitive guidance. Clinical experience in NIS remains limited but emerging, with case reports and small case series describing successful inflammatory control and corticosteroid-sparing effects, particularly with tofacitinib and upadacitinib in refractory disease. Real-world comparative data suggest an association between JAK inhibitor use and a lower incidence of uveitis compared with anti-TNF-α agents in spondyloarthropathies (HR = 0.63; 95% CI, 0.42–0.95), consistent with a potential class effect (183).

Given their oral administration, rapid onset of action, and ability to inhibit multiple cytokine pathways simultaneously, JAK inhibitors represent promising options for patients with NIU. Nevertheless, the safety profile of these agents continues to evolve and appears to be both dose- and agent-specific, underscoring the need for continued pharmacovigilance and longer-term, drug-specific safety data.

### 3.2 Dihydroorotate dehydrogenase inhibitors

Dihydroorotate dehydrogenase (DHODH) is a mitochondrial enzyme essential for *de novo* pyrimidine nucleotide synthesis and is critical for the proliferation of activated T lymphocytes. KIO-100 (Kiora Pharmaceuticals, Salt Lake City, UT, Unted States), formerly known as PP-001, is a potent and highly selective small-molecule DHODH inhibitor developed for local ocular delivery. Compared with leflunomide, KIO-100 demonstrates substantially greater inhibitory potency and target specificity and does not require hepatic bioactivation (184). In a first-in-human, prospective phase 1 open-label clinical trial, a single intravitreal injection of KIO-100 was evaluated in 12 patients with chronic non-infectious posterior segment uveitis (185). The study demonstrated good tolerability, a reduction in intraocular inflammation, and dose-dependent improvements in VA, with regression of macular edema observed in some patients and no drug-related adverse events reported.(185)

Building on these findings, an ongoing multicenter phase 2 open-label study (KLARITY-1; NCT04924184) was initiated to further evaluate localized DHODH inhibition using KIO-104, an intravitreal formulation within the KIO compound family.(186) KLARITY-1 investigates the safety, tolerability, and efficacy of repeated intravitreal injections in patients with macular edema secondary to NIU, retinal vein occlusion, diabetic retinopathy, or post-cataract surgery. The study includes multiple dosing cohorts with administrations every 2 or 4 weeks and is expected to conclude in late 2026 (186). By defining optimal dosing regimens and characterizing clinical response, this study will further clarify the therapeutic potential of site-specific DHODH inhibition for inflammatory and retinal disease-associated macular edema. Although early results are encouraging, longer-term data are required to assess durability of efficacy and optimal patient selection.

### 3.3 Multi-target immune modulator

Dazdotuftide (TRS01, Tarsier Pharma Ltd., Tel Aviv, Israel) is a small-molecule immunomodulator developed for topical ocular use. Its proposed mechanism of action involves modulation of innate immune responses, including a shift in macrophage polarization from a pro-inflammatory (M1) phenotype toward an anti-inflammatory, IL-10-secreting (M2) phenotype (187).

TRS01 ophthalmic solution has been evaluated in non-infectious anterior uveitis in a phase 3, prospective, multicenter, randomized, active-controlled, double-masked study (188). A total of 136 patients were randomized 2:1 to receive TRS01 1% eye drops or prednisolone acetate 1%. At week 4, 48% of patients treated with TRS01 achieved complete resolution of anterior chamber cells, compared with 68% in the prednisolone acetate group (*P* = 0.0311). Clinically meaningful improvement was observed in 64% of TRS01-treated patients versus 89% of steroid-treated patients (*P* = 0.0049). Although TRS01 was inferior to topical corticosteroids in achieving complete anterior chamber cell resolution, it was non-inferior to prednisolone acetate in controlling anterior chamber flare and ocular pain. Importantly, secondary and *post hoc* analyses demonstrated a more favorable intraocular pressure (IOP) safety profile with TRS01, with significantly fewer IOP elevations compared with steroid therapy. Among patients who achieved complete inflammatory resolution, TRS01-treated individuals showed a more favorable IOP safety profile, with smaller changes from baseline and fewer threshold IOP elevations across all evaluated cutoffs (*P* < 0.05) (188). Based on these findings, a subsequent phase 3 trial (Tarsier-04) has been initiated under a U.S. FDA special protocol assessment to further evaluate the efficacy and safety of TRS01 using revised, clinically relevant endpoints. Tarsier-04 is a multicenter, randomized, double-masked, active-controlled study designed to replicate the prior trial with a focus on sustained inflammation control and overall benefit–risk assessment.(189) Collectively, these findings support the further evaluation of dazdotuftide as a non-steroidal topical therapy for non-infectious anterior uveitis, particularly in clinical settings where control of inflammation must be balanced against the risk of steroid-associated IOP elevation, such as in patients with uveitic glaucoma.

### 3.4 Reactive aldehyde species inhibitors

Reactive aldehyde species (RASP) inhibitors, also referred to as aldehyde-sequestering agents, target downstream inflammatory pathways by neutralizing reactive aldehydes generated during oxidative stress. Through this mechanism, RASP inhibition may limit protein modification, autoantibody formation, and NF-κB activation, thereby attenuating inflammatory signaling without directly suppressing immune cell function (139).

Reproxalap (ADX-102^®^, Aldeyra Therapeutics, Lexington, MA, United States), a topical RASP inhibitor, has been evaluated in non-infectious anterior uveitis. In a randomized phase 2 trial, reproxalap monotherapy, combination reproxalap plus prednisolone, and prednisolone alone demonstrated comparable rates of treatment success (approximately 40%, 44%, and 46%, respectively), with similar improvements in anterior chamber inflammation and time-to-event outcomes. Reproxalap was non-inferior to topical corticosteroids and was associated with a shorter mean time to treatment success in the monotherapy arm. Importantly, reproxalap was well tolerated, with mild ocular irritation as the most common adverse event, and without the intraocular pressure elevation observed in the steroid-treated group (190).

Reproxalap was subsequently evaluated in the phase 3 SOLACE trial (NCT03131154), a randomized, double-masked, vehicle-controlled study in patients with acute non-infectious anterior uveitis.(191) The study did not meet its primary or secondary efficacy endpoints, primarily attributable to high rates of disease resolution in the vehicle-treated arm. Following these results, further development of reproxalap for non-infectious anterior uveitis was discontinued, and clinical efforts were redirected toward other ocular inflammatory indications, including dry eye disease, allergic conjunctivitis, and proliferative vitreoretinopathy (192, 193).

Overall, available clinical data suggest that RASP inhibition represents a novel, non-steroidal therapeutic approach for anterior uveitis, with a safety profile characterized predominantly by mild, self-limited ocular adverse events. While efficacy appears more modest than that of corticosteroids in acute disease, RASP inhibitors may hold particular relevance as adjunctive or steroid-sparing options, especially in patients for whom minimizing corticosteroid exposure or intraocular pressure elevation is a clinical priority. Further optimization of trial design and patient selection will be essential to better define their role in the management of ocular inflammation (139).

## 4 Cellular and immune network modulation

### 4.1 T-cell co-stimulation modulation

Abatacept (Orencia, Bristol Myers Squibb, Princeton, NJ, United States) is a recombinant fusion protein consisting of the extracellular domain of human cytotoxic T-lymphocyte–associated antigen 4 (CTLA-4) linked to the modified Fc portion of human immunoglobulin G1. T-cell activation requires two distinct signals delivered by antigen-presenting cells: recognition of antigen in the context of major histocompatibility complex molecules, and a costimulatory signal mediated through CD80 or CD86 binding to CD28 on T cells. Abatacept selectively binds to CD80 and CD86, thereby blocking this second, costimulatory signal and preventing full T-cell activation and downstream immune responses (194). Through this targeted mechanism, abatacept has become an established therapy for several immune-mediated diseases. It is approved for the treatment of moderate-to-severe RA in adults, and for polyarticular JIA, active PsA, and prevention of acute graft-versus-host disease in patients undergoing allogeneic hematopoietic stem cell transplantation. The drug can be administered IV or, in adults, by the SC route.

Beyond its approved indications, abatacept has generated growing interest as an off-label option for refractory immune-mediated conditions. Given the central role of T-cell costimulation in many inflammatory pathways, inhibition of the CD28–CD80/86 axis has been explored in difficult-to-treat systemic and ocular inflammatory diseases. The first reported use of abatacept in uveitis was a 2008 pediatric case of a 16-year-old girl with PsA–associated uveitis refractory to multiple immunosuppressive and biologic agents (195). IV abatacept led to rapid control of ocular inflammation, allowed tapering of steroids and immunosuppressants, and maintained good VA with sustained disease control over 18 months (195). Subsequent data on abatacept in uveitis come from JIA-associated uveitis, mainly as case reports, small series, and retrospective studies, showing frequent remission and marked flare reduction in severe anti-TNF-refractory cases, and in some patients uveitis improved despite persistent or flaring joint disease (196–198). Long-term efficacy of abatacept has been reported in a small case series of three patients with refractory idiopathic uveitis treated with monthly IV abatacept. All achieved remission and discontinued systemic corticosteroids after a mean of 30 weeks, with disease control maintained over prolonged follow-up of 42, 33, and 18 months, respectively, and only mild, infrequent flares that were managed with topical therapy.(199) However, in a larger multicenter retrospective study of 21 patients with JIA-associated uveitis, uveitis inactivity was achieved in 11 (52%), but subsequently recurred in 8, remained persistently active in 10, and sustained response was uncommon. Only three patients receiving systemic corticosteroids and immunosuppressive agents were able to taper these treatments (200). In a national cohort of 125 JIA-associated uveitis patients, 4 (3.2%) received abatacept, and 2 discontinued because of loss of efficacy (92). In another series of alternative biologics, 2 of 3 children failed abatacept before achieving disease control with other agents (60).

A multicenter retrospective study evaluated abatacept as a first-line biologic agent in severe JIA-associated uveitis refractory to at least 6 months of methotrexate and compared outcomes with its use after prior anti-TNF therapy. Among 35 treated patients, 31 completed 12 months of follow-up, with over half achieving clinical remission; the frequency of uveitis flares significantly decreased in both first-line and second-line groups, and time to relapse was comparable (201). In 2024, the first prospective evaluation of abatacept in posterior uveitis investigated SC abatacept in 15 patients with BSCR and demonstrated significant improvement in vitreous haze, angiographic inflammation scores, and choroidal thickness, although two patients met criteria for treatment failure during follow-up (202). Abatacept has also shown efficacy in uveitis associated with CTLA-4 deficiency. Case reports describe rapid resolution of multifocal choroiditis and improvement in vision in adults (203), and sustained remission of intermediate uveitis in children with CTLA-4 haploinsufficiency (204). An open-label, 2-year, phase 2 study is evaluating abatacept (5 or 10 mg/kg every 4 weeks) in adults with NIU refractory to prior therapies (NCT01279954). The planned enrollment is 20 patients, with outcomes including changes in VA, vitreous haze, and reduction of corticosteroids or other immunosuppressive agents. Although the study is listed as completed, detailed results have not yet been published (205).

Evidence for abatacept in scleritis remains scarce. It was first reported in a retrospective multicenter study of 14 refractory NIS cases. In this series, abatacept was administered subcutaneously in three patients (4 eyes) with anterior scleritis associated with RA, mainly for control of systemic disease. All treated eyes had active anterior diffuse scleritis at baseline, and two of the three patients had no scleritis relapse during the 12 months after starting abatacept; both of these patients received concomitant methotrexate. In contrast, the remaining patient, who had bilateral scleritis and was treated with abatacept monotherapy, did not demonstrate clinical improvement.(95)

#### 4.1.1 Safety considerations and summary

Across published studies, including prospective trials, case reports, small series, and retrospective cohorts, abatacept has shown a generally favorable safety profile in uveitis and scleritis, with most reports describing no serious drug-related adverse events or infusion reactions. When adverse events occurred, they were usually mild and transient, such as headache, fatigue, weight gain, diarrhea, skin reactions, or oral mycosis (200–202, 206). Overall, abatacept appears to be well-tolerated in ocular inflammatory diseases, although interpretation is limited by small sample sizes and heterogeneous study designs. Although current evidence remains limited, emerging clinical data suggest that abatacept may offer meaningful disease control in specific conditions or in carefully selected patients who are refractory to conventional immunosuppressive or biologic therapies.

### 4.2 B-cell–targeted therapies

Rituximab (Rituxan^®^, Genentech/Biogen, South San Francisco, CA, United States/Cambridge, MA, United States) is a chimeric murine-human mAb targeting the CD20 antigen expressed on naïve and memory B lymphocytes, leading to prolonged B-cell depletion and modulation of antibody-mediated and cellular immune responses. This depletion typically lasts 6–12 months and spares long-lived plasma cells and hematopoietic stem cells, resulting in reduced circulating IgG and IgM levels and suppression of pathogenic B-cell activity. These mechanisms highlight the pivotal role of B cells in the pathogenesis of autoimmune and inflammatory diseases. Rituximab is approved by the FDA for multiple oncologic indications, including non-Hodgkin B-cell lymphoma, chronic lymphocytic leukemia, and pediatric mature B-cell malignancies, as well as for non-oncologic immune-mediated diseases such as RA, granulomatosis with polyangiitis (GPA), microscopic polyangiitis, and pemphigus vulgaris. Beyond these approved indications, rituximab has gained increasing use in severe and refractory NIU and NIS, especially in patients with associated systemic autoimmune disorders or disease resistant to corticosteroids, conventional immunosuppressive agents, and anti-TNF-α therapies.

Evidence for rituximab in NIU has largely been derived from case reports and small retrospective case series. The first published report appeared in 2007 as a single case of refractory anterior uveitis. In this report, a 49-year-old woman with bilateral chronic non-infectious anterior uveitis unresponsive to corticosteroids and conventional immunosuppressive therapy was treated with rituximab at 375 mg/m^2^ weekly for 4 consecutive weeks. Following treatment, ocular inflammation stabilized, CME improved, and VA increased, with sustained disease control maintained over a 12-month follow-up period.(207) Among NIU subtypes, VKH disease represents one of the conditions with comparatively more accumulated clinical experience supporting rituximab use. Available evidence overall demonstrates favorable clinical responses in refractory cases. Across published reports, rituximab has been associated with rapid suppression of intraocular inflammation, with clinical improvement often observed after the first one to two infusions, followed by sustained disease quiescence. Several case reports and small series have described sustained disease control with stabilization or improvement in visual acuity (208–211). In one reported case, a patient received four doses of rituximab (1 g) through 16 months and remained relapse-free through 34 months of follow-up, suggesting the potential for durable remission in selected cases. Retrospective series further support high remission rates, significant improvement in visual outcomes and choroidal thickness, and meaningful corticosteroid-sparing effects (212, 213). The largest retrospective series to date included 24 patients with refractory NIU and NIS, of whom 16 had VKH disease. Rituximab was administered intravenously at a dose of 1,000 mg (or 375 mg/m^2^ in pediatric patients) on days 1 and 15, with repeat dosing at 6 months. Disease control was achieved after three infusions in 62.5% of patients, whereas 37.5% required additional treatment cycles, with some patients receiving up to seven total infusions. Overall, rituximab induced and maintained disease quiescence, with significant improvement in VA over a mean follow-up of 41 months, and all patients remained flare-free at last follow-up. Corticosteroid use was markedly reduced, with 87.5% of patients able to discontinue steroids completely, supporting rituximab as an effective option for severe, treatment-refractory VKH disease (214). After VKH disease, JIA-associated uveitis represents the second largest body of evidence for rituximab use in NIU. In contrast to VKH, the available data in JIA-associated uveitis appear more heterogeneous. Retrospective studies and small case series suggest that rituximab can induce uveitis inactivity and reduce corticosteroid requirements in a subset of patients with severe disease refractory to conventional immunosuppressive and anti-TNF-α therapies. However, relapses and treatment failure have been reported, with some patients requiring repeated courses or demonstrating persistent ocular activity, and others discontinuing therapy due to inadequate control of joint disease (43, 215–217). Evidence for rituximab in Behçet’s uveitis remains limited but encouraging. Available data include early case reports and a single randomized pilot study—the only randomized evidence for rituximab in non-infectious uveitis—demonstrating significant reductions in disease activity indices, improvement in posterior uveitis, and sustained remission in some patients for up to 24 months (218, 219). In MS-associated uveitis, evidence from case reports and small retrospective series suggests that rituximab can effectively control intraocular inflammation and retinal vasculitis while stabilizing neurological disease, with improvements in VA, inflammatory scores, and reduced relapse rates (220–222). However, unsuccessful treatment has been reported in at least one case in which high-dose rituximab led to severe hypogammaglobulinemia complicated by bacterial pneumonia, necessitating drug discontinuation (19). Beyond these conditions, rituximab has also been reported to achieve favorable outcomes across a range of other ocular inflammatory diseases, including uveitis or retinal vasculitis associated with GPA, sarcoidosis, systemic lupus erythematosus, HLA-B27–associated disease, RA, type 2 essential cryoglobulinemia, diffuse subretinal fibrosis and uveitis syndrome, BSCR, and idiopathic NIU (222–231). In contrast, unfavorable outcomes have also been reported. A recent case described exacerbation of BSCR occurring after the first dose of rituximab, with worsening choroidal inflammation, increased choroidal thickness, subretinal fluid, and retinal dysfunction on multimodal imaging (232).

Additionally, rituximab has also been investigated for its potential role in paraneoplastic retinopathy and autoimmune retinopathy. Overall, the available evidence, derived from case reports, retrospective series, and a small prospective phase 1/2 trial, demonstrates heterogeneous outcomes (233–246). Across studies, rituximab was more frequently associated with stabilization or slowing of visual deterioration rather than marked visual recovery, particularly when initiated early in the disease course. While some patients showed functional or electrophysiologic improvement, others experienced continued progression or treatment failure, highlighting variable efficacy in these retinal autoimmune conditions (233–246).

Evidence supporting the use of rituximab in NIS is relatively substantial, particularly in GPA. Across multiple case reports and retrospective series, rituximab has shown high efficacy in inducing remission of refractory GPA-associated scleritis, often with rapid clinical improvement, reduction in ANCA titers, and significant corticosteroid-sparing effects (247–260). The largest retrospective cohort evaluated 37 patients with ocular GPA, including 20 with scleritis and 17 with orbital disease, treated with rituximab. Remission, defined as inactive disease on ≤7.5 mg/day of prednisolone with or without maintenance therapy, was achieved in 86% of patients, with comparable outcomes between scleritis and orbital involvement as well as between localized and generalized disease. Although relapses occurred in approximately half of patients, re-treatment with rituximab was consistently effective, supporting its role in both induction and re-induction of remission (260). However, paradoxical worsening and treatment-related complications have also been reported, including post-infusion exacerbation of scleritis, posterior scleritis with orbital inflammation, macular edema, and rare severe ocular or orbital flares occurring shortly after rituximab administration. In some cases, protocol modification, intensified corticosteroid coverage, or switching to alternative biologic agents was necessary to achieve disease control (261–265). Beyond GPA, rituximab has also been reported to achieve favorable outcomes in scleritis associated with other conditions, including RA, relapsing polychondritis, ANCA-associated vasculitis, IgG4-related disease, Sjögren syndrome, necrotizing scleritis following pterygium excision, and idiopathic scleritis, with evidence derived primarily from case reports and retrospective case series (95, 266–277). A prospective phase 1/2 randomized, double-masked trial comparing two rituximab dosing regimens (500 vs. 1,000 mg administered on days 1 and 15) further supports its efficacy in refractory NIS (278). In this dose-ranging study of 12 patients, nine achieved a clinical response by 24 weeks, with improvements in scleritis activity, pain, and patient and physician-reported health assessment, although most required re-treatment to sustain disease control. Early peri-infusional inflammatory flares were observed but were effectively mitigated with corticosteroid prophylaxis, and no significant differences in efficacy or safety were detected between dosing arms (278). Subconjunctival rituximab has also been explored in a small case series of refractory NIS; however, administration of low-dose subconjunctival rituximab (2.5–7.5 mg) resulted in minimal to no improvement in clinical signs or ultrasonographic findings over 8–10 months of follow-up (279).

#### 4.2.1 Safety considerations and summary

Overall, rituximab has been generally well tolerated in NIU and NIS, with most adverse events being mild and infusion related, including pruritus, flushing, rashes, urticaria, anxiety, leukopenia, headache, tachycardia, and hypotension (219, 229, 239, 241, 252, 260). These events were typically manageable with infusion rate adjustment or corticosteroid premedication. However, infectious complications remain the most important safety concern, particularly with repeated dosing and concomitant immunosuppression. In a large retrospective cohort of ocular GPA, infections were the most frequent adverse events, with approximately 8% of patients requiring hospitalization and a subset developing hypogammaglobulinemia requiring intravenous immunoglobulin (260, 270). Other reported infections across studies included bacterial pneumonia, bacterial skin infections, herpes zoster, herpetic mouth ulcers, fungal infections, recurrent sinus or urinary tract infections, and COVID-19 (19, 95, 219, 222, 225, 228, 229, 241, 252, 276). Severe infections have been reported rarely, including a fatal case of Pneumocystis jirovecii pneumonia following rituximab and systemic corticosteroids for posterior scleritis, as well as a case of acute retinal necrosis developing during rituximab maintenance therapy for RA-associated scleritis (280, 281).

Despite accumulating clinical experience, the current evidence supporting rituximab in NIU and NIS remains predominantly derived from non-randomized, retrospective studies and small prospective cohorts. In NIU, published data are limited to selected disease entities, whereas experience with rituximab in scleritis appears more extensive and is generally associated with favorable clinical responses. Collectively, these findings suggest that rituximab may represent a therapeutic option in carefully selected cases of refractory ocular inflammation, while underscoring the need for further prospective, controlled studies.

## 5 Conclusion

In recent years, expanding insight into immune mechanisms underlying ocular inflammation has led to the development of multiple targeted therapeutic strategies beyond conventional corticosteroids, immunosuppressants, and anti-TNF-α therapies. Collectively, the emerging therapeutic agents reviewed herein represent diverse mechanistic classes, including cytokine blockade, intracellular signaling inhibitors, cellular immune modulators, and locally delivered small-molecule therapies. Overall, the strength of clinical evidence remains heterogeneous. For most agents, published data are derived predominantly from case reports, small case series, and retrospective observational cohorts, reflecting both disease rarity and clinical heterogeneity. Prospective and randomized evidence remains limited but is gradually emerging for selected pathways. When assessed using the GRADE framework, the certainty of evidence across emerging therapies ranges from very low to moderate, reflecting these underlying limitations in study design and data maturity.

Among cytokine-targeted therapies, IL-6 receptor blockade with tocilizumab has one of the most robust clinical evidence bases in NIU, supported by prospective trials and multicenter cohorts. Consistent benefit has been observed in UME, retinal vasculitis, and selected cases of NIS, with supportive data also emerging in pediatric uveitis. In contrast, evidence for sarilumab remains limited to a single phase 2 randomized trial. IL-1 blockade has been most frequently evaluated in Behçet’s uveitis, where observational data suggest improvements in inflammatory control and corticosteroid reduction across both NIU and NIS. Evidence for canakinumab remains limited, primarily as second-line or intra-class switching therapy, while gevokizumab did not meet phase 3 primary endpoints despite signals of potential benefit in secondary analyses and small NIS studies. Clinical evidence for IL-17 blockade in NIU remains limited and inconsistent. Phase 3 trials of secukinumab failed to meet primary efficacy endpoints, with subsequent data confined largely to anti-TNF-refractory case series and heterogeneous real-world outcomes. Evidence for ustekinumab, IL12/23 inhibitor, is limited but suggests potential benefit in carefully selected refractory NIU cases.

Among intracellular pathway modulators, oral JAK inhibition has accumulated increasingly persuasive real-world evidence across diverse NIU/NIS phenotypes and includes randomized trial signals in NIU for select agents. Together with convenient oral administration, rapid onset of action, and multi-cytokine pathway coverage, this class appears among the most clinically translatable emerging options, provided safety monitoring and patient selection are appropriate. Other small-molecule and local therapies, including DHODH inhibitors, RASP inhibitors, and multi-target immune modulators, remain in early clinical development but introduce important concepts in site-specific immunomodulation and steroid-sparing topical therapy in NIU.

Within cellular and immune network modulation, abatacept has been reported most often in JIA-associated uveitis, where observational data suggest flare reduction and corticosteroid-sparing effects in a subset of anti-TNF-refractory patients, although sustained inactivity is inconsistent. Evidence in NIS remains limited and insufficient to define its role beyond selected refractory cases. Rituximab has the most substantial clinical evidence base among emerging biologic therapies for NIS, particularly in GPA. It has also been widely studied in refractory NIU, demonstrating the most consistent favorable outcomes in VKH disease and Behçet’s uveitis, while responses in JIA-associated uveitis, paraneoplastic retinopathy, and autoimmune retinopathy appear more heterogeneous. However, infectious complications and disease relapse necessitating repeat dosing remain important considerations in long-term management. Importantly, safety observations across emerging non-TNF-α biologic pathways have been generally reassuring. In contrast to anti-TNF-α therapy, reactivation of latent tuberculosis and demyelinating complications have not been consistently observed in ophthalmic studies to date, although routine screening remains essential.

Taken together, among emerging targeted therapies, tocilizumab, JAK inhibitors, and rituximab currently have the most substantial and clinically supportive evidence for the treatment of NIU and NIS. Based on the distribution of reported cases and the breadth of available evidence, tocilizumab appears to have the most extensive clinical experience among these agents, particularly in NIU and uveitic macular edema. Its use has been most frequently reported in idiopathic uveitis, JIA-associated uveitis, BSCR, and Behçet’s uveitis. JAK inhibitors represent the next most supported class, with accumulating evidence in JIA-associated and idiopathic uveitis. In comparison, rituximab has demonstrated favorable outcomes in more selected disease subsets, including VKH and GPA-associated scleritis. Notably, most of these therapies have been evaluated as third- or fourth-line options in patients with refractory NIU and NIS, typically following inadequate response or intolerance to conventional immunomodulatory and anti-TNF-α therapies. Therefore, their use in clinical practice should be guided by the currently available evidence, particularly in refractory cases with similar disease characteristics and prior treatment exposure.

## 6 Future perspectives

Despite rapid therapeutic expansion, several unmet needs remain. First, the current evidence base for most emerging therapies is still limited by small sample sizes, retrospective study design, and marked disease heterogeneity, underscoring the need for adequately powered randomized controlled trials to validate early efficacy signals and define optimal positioning within treatment algorithms. Second, head-to-head comparative studies, particularly against anti-TNF-α agents, are required to clarify relative efficacy, durability of remission, and corticosteroid sparing capacity across biologic and targeted therapeutic classes, while also improving understanding of which therapies are most effective for specific disease subtypes. Last, long term safety surveillance, pharmacovigilance, and cost effectiveness analyses remain essential as these agents enter broader clinical use. Taken together, while emerging agents expand the therapeutic armamentarium for NIU and NIS, their use remains largely reserved for refractory disease, guided by mechanism, systemic associations, and individual risk profiles.
